# Understanding the lived-experience and support-needs of people living with antimicrobial resistance in the UK through interpretative phenomenological analysis

**DOI:** 10.1038/s41598-024-53814-6

**Published:** 2024-02-10

**Authors:** Ryan A. Hamilton, Benjamin Lond, Lucina Wilde, Iain Williamson

**Affiliations:** 1 School of Pharmacy, De Montfort University, Leicester, UK; 2 Department of Psychology, De Montfort University, Leicester, UK

**Keywords:** Patient education, Health services, Human behaviour, Antimicrobial resistance, Infectious diseases, Quality of life

## Abstract

In the UK nearly 54,000 infections were caused by serious resistant bacteria in 2022 but there is a lack of evidence regarding the long-term impact on patients’ lives nor what support they need. This research aimed to answer the question: “What are the key elements of experience and support needs of people living with AMR in the UK?”. In-depth semi-structured interviews were undertaken with nine people who had been living with resistant infections or colonisation for 12-months or longer. Interpretive Phenomenological Analysis was used to study the accounts and illustrate individuals’ experiences and support-needs. Participants experienced marginalisation and isolation but also empowerment; described across three major themes: (1) I live in fear and stigma: The long-term impact of AMR; (2) I am battling on my own: A journey toward self-advocacy; and (3) I like to share my story: The role of AMR communities. All participants perceived a lack of knowledge, information, and support from clinicians; difficulties accessing reliable and understandable information; and lack of understanding from family and friends. Charities and online groups provided support with coping with their situation and improving mental health and wellbeing. Understandable and relatable information regarding the science of AMR, transmission, prevention, and living with AMR needs to be provided by clinicians and healthcare services around the time of diagnosis to readily available after diagnosis.

## Introduction

Globally, more than 1.25 million people are estimated to die annually from antimicrobial resistant (AMR) infections, with it being a contributing factor in an additional 3.7 million deaths^1^. In the UK alone, in 2022 it was estimated that 53,985 infections were caused by serious AMR bacteria^2^. Infections caused by AMR bacteria necessitate the use of antimicrobial therapies that have higher rates of adverse effects and are more costly, yet still these infections result in greater mortality when compared to drug-susceptible infections^3–5^. Increasing rates of colonisation (asymptomatic carriage) can also lead to local transmission and outbreaks, which poses a risk to patients who develop infections in the short and long term^6^. The psychosocial impact, however, on people experiencing AMR infections or living with chronic colonisation with AMR organisms is poorly understood.

When attending hospital, individuals who are known to be colonised with multi drug- or extensively drug-resistant (MDR and XDR, respectively) organisms are typically isolated in single rooms and given the last slots for procedures^7^, with staff wearing enhanced personal protective equipment (PPE)^8–10^. Studies have shown that many health and care professionals (HCPs) lack confidence in both preventing transmission and the management of patients with AMR^11^. It is unsurprising that this has a negative impact on the care that patients receive (both medically and psychologically) and negatively affects their experience of healthcare, which has been the focus of most research to-date^12–16^. A systematic review of the literature by Rump et al.^8^ mapped the existing literature against the capabilities postulated by Nussbaum^17^ and proposed that living with AMR impacts on an individual’s *bodily health; emotion; practical reason; affiliation; being able to laugh, play, and to enjoy recreational activity;* and* having control over one’s environments.*

Emerging evidence indicates that patients lack an understanding of what AMR is, yet they also report significant psychological and social impact upon receiving a diagnosis of AMR including stigmatisation and associated distress as well as exclusion from participation in social relationships with friends and family^8,18–21^. Little work has been conducted in the UK^22^ and no research has described the longer-term consequences of living with AMR nor what support and advice these individuals require.

The central research question that this research aims to answer is: “What are the key elements of experience and support needs of people living with AMR in the United Kingdom?” Through the use of in-depth interviews, this research sought to provide a detailed understanding of people’s experiences of living with AMR in the UK; elucidating the long-term impact on their daily lives, health, and overall wellbeing. Moreover, this research fills a significant gap in the evidence-base regarding the support and advice these individuals require.

## Methods

### Study design

Because of the focus on lived experience, a phenomenological qualitative approach was chosen for this study, specifically interpretative phenomenological analysis which has been developed for in-depth reporting and interpretation of in-depth data collected from a small number of relatively homogenous participants, typically ten or fewer^23^. Data were gathered through in-depth semi-structured interviews^24^ with people living with AMR infection(s) and/or colonised with an AMR microbe in the UK. Because ethical approval was gained and data were collected under research recommendations developed in response to the COVID-19 pandemic, all interviews took place by phone or online following recommended adaptations for virtual interviewing^25^.

### Participants

Purposive sampling was used in this study. To be eligible for participation, individuals must have been diagnosed with an AMR infection, or colonised with an AMR microbe, for twelve months or longer to study longer term impact, be aged 18 or above, and be resident and receiving their care and treatment in the UK. Nineteen individuals expressed an interest in the study, however ten did not subsequently proceed to be interviewed. The final sample comprised seven females and two males aged between 26 and 78 years, who had been living with AMR for between approximately one and eight years. The ethnicity of participants, infection type, and the year of initial diagnosis are included in Table 1. All participants reported active AMR infections; no patients reported asymptomatic carriage without any infections since diagnosis. Of those living with urinary tract infections, *Escherichia coli* and *Citrobacter* species were identified as the causative organisms whereas MRSA (methicillin resistant *Staphylococcus aureus*) was reported by participants experiencing skin and skin-structure infections.

**Table 1:** Participant demographics.

Participant	Sex	Age (years)	Ethnicity	Year of AMR diagnosis	Type/source of infection	Interview via	Length of interview
F40	Female	40	White British	2018	Urinary tract	Microsoft Teams	1 h and 35 min
F50	Female	50	White British	2014	Urinary tract and skin and skin-structure	Microsoft Teams	1 h and 10 min
F58	Female	58	White British	2017	Urinary tract	Microsoft Teams	54 min
F48(1)	Female	48	White British	2019	Urinary tract	Telephone	1 h
F52	Female	52	White British	2017	Urinary tract	Microsoft Teams	59 min
F61	Female	61	White British	2000	Urinary tract	Telephone	1 h and 20 min
F48(2)	Female	48	Other white	2021	Urinary tract	Microsoft Teams	1 h and 18 min
M78	Male	78	White British	2016	Skin and skin-structure and osteomyelitis	Telephone	1 h and 22 min
M26	Male	26	Black Caribbean	2019	Skin and skin-structure	Microsoft Teams	30 min

### Materials

A study flyer, participant information sheet, and consent form were produced for the study as well as an interview schedule and short demographic questionnaire. These latter two resources are available in the “Supplementary Materials”.

### Procedure

A study flyer and a study web-page were developed to inform potential participants about the study. These resources included information on the aims of the study, eligibility criteria and what participation would entail. The study was advertised online on social media platforms which are often used by people living with antimicrobial resistant infections, including Twitter and specific Facebook support groups. The study flyer and link to the webpage were also disseminated on the mailing list of an Antibiotic Research UK (ANTRUK) patient support group. Interested individuals were asked to contact the research team to express an interest in the study. A member of the research team responded to all enquiries with a Participant Information Sheet, a voluntary Demographic Form and a Consent Form. Once consent forms were received, interviews were arranged at a date and time convenient for the participants.

One-to-one, semi-structured interviews were conducted in 2022. Interviews took place either online via Microsoft Teams, or telephone, depending on the participant’s preference. Interviews began with building rapport followed by an open, orientating question: “Can you tell me about your antibiotic resistant infection(s) that you currently have or have had previously”. This allowed participants to convey their understanding of their condition and identify the experiences which were pertinent to them, which were then discussed in more depth throughout the interviews. A semi-structured interview schedule was used to ensure the research questions were addressed whilst allowing flexibility to capture spontaneous or additional material that was raised by participants. Interviews lasted between 30 min and 1 h and 35 min (mean = 67.5 min; median = 70 min).

### Ethics

Ethical approval was granted by De Montfort University’s Research Ethics Committee (Ref: 447782). Informed consent was obtained either through the completion of a written consent form, or verbal consent was taken at the beginning of the interviews when written consent was not possible. Participants were reminded of their right to withdraw. All participants provided informed and explicit consent to the recording of the interviews and anonymity of the data was ensured. All interviews and data analysis were undertaken in line with the ethics-approved protocol and relevant university and UK guidelines and regulations.

### Data analysis

Interviews were audio-recorded and transcribed verbatim. Any identifiable information was anonymised at the point of transcription to protect participants’ identities. As the aims of the study were to understand the lived experience of people living with AMR, Interpretative Phenomenological Analysis (IPA) was adopted as the most appropriate methodology for data analysis. As mentioned above, an IPA approach focuses on the distillation and interpretation of in-depth experiential data collected from a small sample of participants experiencing a similar situation. Saturation is not an aim of IPA and is not claimed by the authors^23^.

As outlined by Smith et al.^23^, each interview was first read and re-read until the researchers were fully ‘immersed’ in each individual’s account. This was followed by the development of initial themes by noting linguistic, descriptive and conceptual notes on a line-by-line basis. These initial themes were abstracted and integrated to identify connections, resulting in the development of case-specific major themes. In keeping with the idiographic sensibility of IPA, each account was interpreted separately before moving onto the next to ensure the individual themes captured the participant’s individual account. The team then collated, developed and refined a series of major themes (each with constituent sub-themes) that captured common elements of shared experience amongst the sample. These form the basis of the analysis presented. For those interested in either the data collected and/or the transparency and rigour of the analytic processes used, we have provided full shared theme tables in the supplementary materials accompanying this article (Supplementary Table S1).

### Ethics approval and consent to participate

Ethical approval was granted by De Montfort University’s Research Ethics Committee (Ref: 447782). Informed explicit consent was obtained from each participant. See methods section for more detail.

## Results

Three major themes are presented:

1. ‘*I live in fear and* stigma’: The long-term impact of AMR;
2. *‘I am battling on my own*’: A journey toward self-advocacy;
3. ‘*I like to share my story*’: The role of AMR communities.

**Figure 1:**
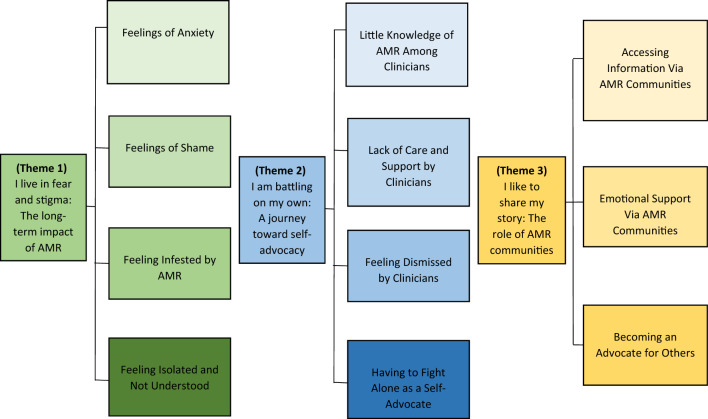
Thematic map illustrating three major themes and subthemes of living with antimicrobial resistance.

Each theme is comprised of several subthemes (see Fig. 1) which are narratively described below alongside identified support-needs. These themes highlight some of the distinct anxieties and internalised stigma felt by people living with AMR, some of which were compounded by their experiences of clinical care and support. Subsequently, individuals spoke of ‘fighting alone’ to manage their condition and situation, while using various online groups and charities to help access health-related information, emotional support, and advocate for others affected by AMR. For each subtheme we have included one indicative quotation from the data corpus. A full set of relevant quotations are included Supplementary Table S1.

### I live in fear and stigma: the long-term impact of AMR (Theme 1)

This theme and its four constituent subthemes outline the psychological impact felt by individuals with AMR which were largely defined by chronic feelings of anxiety, shame, social isolation and a sense of being ‘infested’.

### Feelings of anxiety

Individuals voiced feelings of anxiety that were embedded in fears for the future, including: declining health, limited treatment options, and even death. These fears elicited significant stress and low mood, as some individuals felt resigned to the belief that they would not survive to an old age and lived in worry of future health complications caused by infections that might necessitate drastic treatment, such as organ or limb removal. Participants wanted to understand their diagnosis and prognosis, and how future infections may be treated differently. This information could alleviate anxieties borne out of uncertainty.

*I’m very scared that I’m at the end of the road with the type of antibiotic I I can take.... I often think of I got a shelf life* (F-40)

### Feelings of shame

Several individuals reported instances where they felt shamed by family members, friends, or clinicians; including cases where individuals felt that they were being accused of giving themselves an AMR organism, or made to feel like a nuisance by clinicians for their help-seeking behaviour. Individuals reported how friends and clinicians were particularly cautious of them and anything they touched, eliciting feelings of humiliation and ostracization. Thus, the wider public and clinicians need a greater understanding of infections, AMR and its dissemination so that misconceptions around these do not result in stigma and shame for people living with AMR.

*They’re* [Some of the participant’s friends] *afraid about the contagious, you know this feeling of not being very comfortable like I used to be with my friends. If I’m round someone’s house, I see that they could be just watching my every move.* [M-26]

### Feeling ‘infested’ by AMR

Some individuals represented microorganisms as an anthropomorphised threat that vied to “take control”, “cause trouble”, and “make homes” inside of the person. Such language serves to convey a sense of bodily violation which individuals with AMR may experience as an intruder that undermines bodily autonomy; reinforced by other peoples’ apparent fears of contagion. Individuals feared spreading AMR to others and voiced a lack of control and low confidence in their own bodies. This highlights the need for improved knowledge amongst the general public regarding microorganisms (both commensal and pathogenic), infection, AMR and transmission.

*These things that live in me, they tend to say ‘we’re coming out to play at five o’clock to cause you pain and havoc and to ruin your life’.* [F-40]

### Feeling isolated and not understood

Individuals recalled experiences of social isolation following infection or colonisation with AMR organism(s), describing the need to cancel planned social gatherings and stay home due to poor health. This presented an additional burden as individuals felt guilty and an inconvenience to others due to their relative inability to be a ‘good’ friend and/or reliable family member. Some felt that others could not understand the particular burdens of living with AMR; leaving individuals feeling further isolated and alone. Interpreting this more broadly, patients need information and support to enable them to engage in social activities and maintain connections with others. Increased awareness and understanding from wider society, particularly loved-ones, could allow patients to discuss their psychosocial realities more openly and alleviate feelings of guilt.

*I’ve had to cancel so many things and I hate cancelling things. I hate being that person. I want to be a robust helper for my husband. I want to be a reliable friend. I want to be a reliable, fun granny. There’s lots of things I want to be, but this stops me in my tracks over and over again. It’s not allowing me to be who I was.* [F-61]

### I am battling on my own: a journey toward self-advocacy (Theme 2)

This theme outlines the healthcare experiences of individuals living with AMR, with four subthemes describing how this experience has driven them to take ownership of their situation, advocate for themselves, and become ‘expert patients’. Although individuals sometimes praised the clinical support they had received, they more regularly discussed its limitations.

### Little knowledge of AMR among clinicians

Most individuals felt that non-specialised clinicians regularly had little knowledge or awareness of AMR and were thus poorly equipped to provide sufficient clinical information or guidance. Some individuals recalled how questions to clinicians went unanswered, and they voiced concerns that clinicians sometimes did not always prescribe suitable treatments due to a lack of knowledge regarding AMR. This suggests that clinicians, particularly those with little infection training, need more knowledge and experience regarding AMR and antibiotics in order to better support people living with AMR.

*I was speaking to my doctor just last week. It’s beyond their remit. They don’t know what to say. They don’t know what to do with me*. [F-58]

### Lack of care and support by clinicians

While recognising that clinicians have pressured jobs, most individuals remarked that consultations were often brief with little or no follow-up information and sometimes a perceived lack of compassion. Care was often seen as disjointed and some individuals felt that when clinicians blindly followed clinical guidelines this limited the care and antibiotic treatments they were offered—e.g., they were prescribed empirical options that would be ineffective for the AMR organism(s) they were colonised with. Several individuals explored self-funded treatment; though this was often unaffordable, especially for those with a limited income or ability to work due to AMR. Participants wanted more tailored information and advice from clinicians and person-centred care specific to them and the organism(s) they are living with.

*A sense of being cared for would be more help and a sense of continuity of care would be more help. Honestly, I feel that with every episode whenever infection episode, I have to start at the beginning and I have to work like the devil.* [F-61]

### Feeling dismissed by clinicians

Most individuals felt that some clinicians were dismissive of their concerns and—on occasion—even dismissed the possibility of infection; with symptoms ascribed to hypochondria, age, or injury. This left individuals feeling unheard and unsupported as they had to convince clinicians of the validity and severity of infection to justify further treatment. This shows that even with sufficient knowledge and training, clinicians may need to be more receptive to the ideas, concerns and expectations of patients living with AMR and to explain clinical decisions to patients, particularly where the decision appears unrelated to infection or AMR.

*I said, ‘I really think I’ve got an infection and I don’t think like the antibiotics have worked’ and they said to me, ‘you’ve just got a bad hip and it’s your age’.* [F-48(2)]

### Having to fight alone as a self-advocate

Individuals often discussed in detail a need to adopt a self-reliant attitude with regards to understanding their condition and situation. Participants described how a lack of support alongside a desire to be informed and improve their health, drove them to undertake their own research through online resources and support networks, yet highlighted barriers to accessing trustworthy information they could understand. Individuals also sought information on the science of AMR, antibiotics and treatment options. This new knowledge empowered individuals to advocate for themselves, taking on the role of an expert patient and taking leadership around their own health and needs. Some individuals reported expanding this advocacy outside of healthcare; supporting others living with AMR and raising awareness to organisations and even politicians.

*So it’s really about the education for me and the stewardship and to make sure patients are heard, so you’re on the right dose, the right antibiotic, for the right amount of time*… *you start to become your own expert really in this because you just so desperately wanted to get better.* [F-40]

### I like to share my story: the role of AMR communities (Theme 3)

This theme and its three subthemes present peoples’ experiences of accessing AMR groups that, in lieu of more formalised clinical support, afforded them access to useful information regarding AMR, provided emotional support, and offered a means through which individuals could establish and advocate for the AMR community more generally.

### Accessing information via AMR communities

Almost all individuals credited various online AMR groups and/or charities for providing valuable information about their condition, including information concerning the use and types of medications, and novel therapies, along with more general advice for living with AMR.

*It’s patient support groups without a shadow of a doubt because you could have an open and frank conversation, and people will say have you tried this particular supplement or, when you’re taking this antibiotic makes sure that you do it after a meal or don’t lie down immediately afterwards*. [F-58]

### Emotional support via AMR communities

Individuals credited AMR communities with facilitating peer support, thus allowing individuals to meet and exchange experiences with others affected by AMR. This was further credited by individuals for enhancing their psycho-emotional wellbeing as they found comfort and understanding by talking to people with similar experiences, while nurturing a sense of shared community among those who may otherwise feel socially isolated (Theme 1).

*I think the people that are living with it are the best people to tell their stories and how it’s affected them and what happened to them*. [F-48(1)]

### Becoming an advocate for others

By attending and volunteering for certain groups, some individuals felt they had become more informed about various aspects of AMR. These individuals discussed helping others with AMR, as well as providing information and advice to friends and family. Individuals appeared to welcome the opportunity to ‘give back’ and support others living with AMR.

*My goal in life is to help people with what I can tell them. […] kind of given me a purpose, I suppose in life again*. [F-50]

Given the benefits voiced by almost all participants within this theme, it is clear that providing information regarding appropriate support and advocacy groups within clinical settings is likely to be beneficial to patients living with AMR who may otherwise be unaware of these resources.

## Discussion

This is the first study to utilise a phenomenological approach to understand the lived-experiences and identify the support-needs of people living with AMR infections and chronic colonisation. This is unique amongst the existing literature, which has focused on shared experiences around the time of diagnosis, rather than individual narratives over a significant time post-diagnosis.

### Support needs

The main aim and novelty of this study is that it focuses directly on understanding the support needs of individuals living with AMR, as well as describing the impact on their daily lives inside and outside of healthcare settings. As well as exploring this with participants through explicit questions, we were able to interpret their broader support needs through interpretive analysis of their whole interviews. While different support-needs were identified within each subtheme, these were all inextricably linked.

Participants expressed frustration at the inaccessibility of clinicians, alongside a perceived lack of knowledge and understanding from non-infection specialist clinicians regarding AMR, which lead them to undertake their own research. Similar perceptions have been reported elsewhere^16,20–22^ which suggests an ongoing need for increased knowledge amongst healthcare professionals and a more holistic, supportive, approach to consultations with these patients—not just from a medical perspective, but increasingly a psychosocial perspective. This will help alleviate feelings of isolation, loneliness, stigma, and having to fight alone as a lack of understanding and support from health professionals may exacerbate this^12,22^. Self-advocacy in other settings has been shown to be beneficial to patient experience^26,27^, but it can also be perceived as counterproductive; creating tensions and barriers by clinicians^27,28^ and patients alike^26,29^. Without improved understanding of AMR and its impact on people, this could potentially create a vicious cycle for those living with AMR.

Ultimately, participants wanted to gain a better understanding of their situation from a health and treatment perspective and wanted more support and advice on how to remain socially active and maintain relationships. Participants raised concerns regarding the quality and accuracy of information and advice currently accessible through websites and online support groups but found these provided much needed emotional support and advice on daily living, which has been reported in previous studies into the lived experiences of those with AMR^20–22^. Other studies into the use of social media platforms by the lay public for infection and antibiotic advice has revealed poor knowledge, attitudes and behaviours with regards to the use of antibiotics^30^, which is concerning given the rise of misinformation during the COVID pandemic that included unsolicited advice recommending self-medication with various antimicrobials^31^. Therefore, research into the quality of advice and information available through online platforms is urgently needed.

Research on other chronic, less-common, and less-understood conditions suggests that professional information and support can alleviate peoples’ feelings of anxiety and uncertainty around their health and alleviates feelings of guilt with regards to their relationships^32,33^. Similarly, our participants also wanted to understand the science relating to their specific infections, AMR, and how to prevent its transmission. There is an unmet need for professional and reliable, yet understandable, information about AMR, infections, preventing the transmission of pathogens, and how to live with AMR. The role of family and friends in understanding and supporting people living with AMR came through strongly in our analysis and has been similarly reported by King et al.^22^ as playing a central role in how people came to terms with being colonised with resistant organisms, thus it will be imperative for information to be sharable with family and friends, to improve their knowledge of these topics. Generally, health literacy amongst the general public is low and creates barriers to people understanding their diagnoses and treatment options^34,35^. AMR is a complex and abstract concept that the general public struggle to understand^36,37^ and is more prevalent amongst people from lower socioeconomic and ethnic minority groups^36^, thus information will need to be clear and understandable for lay-persons and those with lower health literacy from a diverse range of backgrounds. Co-creation of resources with diverse groups of patients, particularly expert patients such as those involved in our research, and the public will be vital for creating accessible, understandable and reliable resources.

The literature highlights how there is often an overreliance on specialist clinicians to discuss AMR, colonisation and infection, resulting in delays and conflicting information^22^. Therefore, these information resources could be used to train healthcare professionals about the experiences of people living with AMR, and in turn can be used by health professionals to support patients at the point of initial diagnosis or first encounter with a patient.

In the UK, when an individual is identified with certain AMR organisms, they should receive communication from a healthcare provider to inform them of this^10,38^. It is not known to what extent this is done in the UK and whether people identified with AMR organisms are signposted to relevant information and support, but the narratives of our participants suggest this is rare. Wiklund et al.^20,21^ described how individuals living in Sweden identified with ESBL organisms received letters with brief information about ESBL and their carrier status but contained no follow-on information or support, whereas Hereng et al.^12^ amended their MDR and XDR information to provide to those outside of hospital settings in France. Sharing good practice internationally would be welcomed and more research in Britain specifically is warranted to measure compliance with this national guidance and medicolegal obligations to adequately inform patients of their diagnoses, but our initial recommendation is that these notifications should provide and signpost people to reliable sources of information and support from the outset.

Finally, participants highlighted the value of having spaces to meet others living with AMR, receive emotional support and have a safe space to tell their stories, and even advocate for others. Formal support groups, provided by charities, were highlighted as being the most useful to our participants. As AMR becomes an increasing problem in society there will likely be a need for national and local support groups for people living with AMR. Professionally run support groups are successful and can be a lifeline for people living with conditions that are uncommon or poorly understood by wider society^32^. Support interventions can range from more relationally focused (buddy schemes, group meetings and forums) to more educational-informational driven as research from other long-term conditions shows how needs and priorities vary.

### Lived experiences

Although the focus of our study primarily was to identify the support-needs of individuals living with AMR, it would be remiss of us not to discuss their lived-experiences, through which these support-needs were identified. The participants involved in this study are undertaking a journey to make sense of their situations and comprehend their future, often alone and in isolation. Although we developed three major themes with eleven subthemes, these were all interlinked with each other through our participants’ accounts, which reemphasises the concept of undertaking a journey and developing from someone with little knowledge and understanding, through to expert patient and advocate. By using IPA, we employed a data-driven interpretive approach yet identified key commonalities with reviews by Currie et al.^7^, who performed an inductive approach with the literature, and Rump et al.^8^, who mapped study findings to Nussbaum’s capability framework^17^. Our research reconfirms previously reported lived experiences of people living with AMR and suggests, interestingly, that little has changed in the past 5-years even in the context of the post COVID-19 pandemic world.

Like Currie et al.^7^ we identified a continuum of embodied and emotional responses with those experiencing recurrent UTI experiencing heightened physical responses, whereas those with chronic carriage or prior infections reporting more emotional and psychological impacts. Our participants also described experiences that aligned with negative experiences of healthcare professionals in relation to their infection and needing to adapt their life to their new situation. These were influenced by their individual social contexts, particularly in relation to the general lack of understanding amongst wider society.

As highlighted in the background of this report, Rump et al.^8^ observed how AMR impacted on a wide range of capabilities originally postulated by Nussbaum^17^. Our study also observed an impact on the same capabilities of *bodily health* (capability 2)*; emotion* (capability 5)*; practical reason* (capability 6)*; affiliation* (capability 7)*, being able to laugh, play, and to enjoy recreational activity* (capability 9)*;* and *having control over one’s environments* (capability 10)*.* Additionally, our research identified impact on *Existential threat* (capability 1)—some participants lived in fear of death from infections and an inability to live a full life; *Bodily integrity* (capability 3)—participants described a loss of control over their own bodies, as if they had been invaded; and *Senses, imagination and thought* (capability 4)—they felt their pain was unnecessary and avoidable, but they also reported not being able to freely express their thoughts and feelings.

### Limitations

On face-value the number of participants may be considered a limitation. An IPA approach, however, eschews large numbers of participants and is more concerned with the individual’s unique experience and journey, rather than broad generalisations across a population, or with the notion of achieving saturation^23^. The length of the interviews is a key strength of this research, lasting 60–90 min each, compared to 10–60 min in other research into the experiences of those affected by AMR^12,20–22^. This allowed in-depth analysis and exploration of each individual’s account which we have presented in the supplementary materials (Supplementary Table S1)—since justice could not be done to these narratives within the main manuscript, and we encourage readers to read these for a greater understanding of the lived-experiences and coping-strategies of individuals living with AMR. It would be remiss, however, to ignore the benefits that a larger sample size could bring, especially given the powerful and deep accounts provided by the participants. Although saturation, representativeness, and generalisability are not the goals of IPA methodology, more participants would undoubtedly reveal a broader range of specific support requirements for people living with AMR. Additionally, larger participant numbers, ideally access from a broader range of sources, could provide a more diverse study population that may identify lived experiences and support requirements that might transfer more widely to the community of interest.

The main limitation of this study is that most of the participants identified as white (n = 7/9) and female (n = 7/9) with urinary tract infections (7/9). This will have influenced the major themes and subthemes developed through the analysis of interview transcripts, and further highlights the need for readers to consider the individual accounts presented in Supplementary Table S1. The large proportion of white females in this study may be partly explained by this demographic being more likely to access the kinds of support groups that were used to facilitate recruitment^39,40^. Women are also more likely than men to develop a UTI^41,42^, including recurrent and chronic UTI, which are predominantly caused by enteric Gram-negative organisms that can develop and acquire multiple drug-resistance mechanisms; this is seen in our study population. Future researchers should develop explicit strategies to access more diverse samples and could focus specifically on those who have had single episodes of AMR infections and otherwise healthy individuals with no infections but who have been identified as chronically colonised, who have been excluded from previous studies^12^. It is likely the lived-experiences and support-needs of these populations will differ from those presented in this research.

## Conclusions

There is a lack of support and guidance for people identified with AMR infections and chronical colonisation, which has long-term negative impact on their health and wellbeing. There is an urgent need for professional, reliable, understandable, and relatable information regarding AMR, as a concept; transmission and prevention; living with AMR and prognosis to be provided at the time of diagnosis and readily accessible outside of healthcare. Support-services and groups provided much needed guidance and information on living with AMR and its implications, but we identified a need for professional support services to be expanded. More research is needed into the accessibility and reliability of information currently provided to people living with AMR, and how such a diagnosis impacts people who are asymptomatically colonised with AMR organisms but have never suffered an infection.

## Recommendations for practice

- Professionally run support groups should be formed and easily accessible to people living with AMR—Replicating successful models for other conditions such as cancer.
- Professional and reliable information regarding the science of AMR and its impact on individuals’ lives and health need to be readily available to patients—This information should be co-designed with patients and consider health literacy and cultural barriers. This information should be shared with friends and family to improve understanding of AMR.
- Health professionals need to be more cognisant of the impacts of AMR on individuals and be able to discuss this complex phenomenon with patients.
- Where available, health professionals should proactively signpost patients to reliable information and professional support groups.

### Supplementary Information

Supplementary Table S1.

Supplementary Information.

## Data Availability

All data generated or analysed during this study are included in this published article and its supplementary information files. More information can be requested from the corresponding author.
