# Supplementary material for: Understanding the lived-experience and support-needs of people living with antimicrobial resistance in the UK through interpretative phenomenological analysis
Source: Sci Rep. 2024 Feb 10;14:3403. doi: 10.1038/s41598-024-53814-6 (PMC10858033; doi:10.1038/s41598-024-53814-6)
Supplement: Supplementary file 1 — Supplementary Table S1. [file 41598_2024_53814_MOESM1_ESM.pdf]

# Supplementary Table S1: Collated Themes Table

Transcribed interviews are given bellow in the form of extracts that align to the major themes and subthemes formed through the data analysis process. Coloured sections align to the major themes and subthemes reported in the main article, whereas white sections provide additional information that was not deemed significant enough to form a new subtheme.

No data has been omitted from this table; it presents the complete transcribed dataset.

| Supplementary Table S1. Collated Themes Table | | | |
|---|---|---|---|
| Major Theme | Subtheme | Participant | Example Quotes |
| **(Theme 1)** I live in fear and stigma: The long-term impact of AMR | Feelings of Anxiety | F-40 | I know I could not wait a year, in that time thinking I could die from sepsis, you know |
| | | | mental health. You know, blown to pieces, really not knowing. Uh, you know what's going to happen to me, even now I I I don't know what to do because I feel like this is horrible and lifelong |
| | | | I don't think I'd be alive if it wasn't for that regime that I'm on. I'm very scared that I'm at the end of the road with the type of antibiotic I I can take. Uh, because if I now start to anytime develop serious side effects from that I I don't know what I can do. I I I don't know what the next step is for me and I, 'cause they won't put me back on antibiotics that I've already had severe reactions to, and that's that's the quandary that I find myself in, is I don't know where else what else there is for me |
| | | | I often think of I got a shelf life. I know it's a bit bizarre to say, uh, because there's, as we know, there's no other treatments at the moment available |
| | | | He had multiple infections and he said it just started from one infection, and his immune system got so low he developed multiple infections. That is my worry. And he had, oh, they're all resistant in the end, there's no treatments until he'd done phage therapy and he's been infection free for four years. Erm, that's a worry, that worries me. |
| | | | my life is going to be completely consumed by being on antibiotics and and I feel like I've actually tried nearly every single one possible |
| | | | I don't know what's going to happen to me. I I can't. I I've often feel that my future is very bleak, that there's no future, I feel like I'm being punished, I mean, if I look at it as spirit in a spiritual sense, sometimes I think what have I done in a past life? Why am I being punished? It's hell on earth |
| | | F-50 | I think it has affected it quite a lot. Uh, so I've got other problems going on. I've also got a really bad back that I'm on tablets for that restricts me from doing a lot of things, but UM in myself it's really dragged me down. Uhm it makes me feel really low and anxious. |
| | | | I would not want to go on holiday abroad now because of my fear of going in hospital and getting the infection, I would not wanna go in a hospital abroad or even, you know, getting the infection whilst on the plane and stuff like that so. Uh, you know, we have little holidays around England and you know, and those sort of things are really precious to me |
| | | | whenever we go on holiday now I have to research where the hospitals are up to make sure I'm at least an hours distance driving distance to a hospital and 'cause I'm just paranoid now that you know something terrible is going to happen |

| | | | |
|---|---|---|---|
| | | | it's made me very, very anxious and I never want to go out on my own. And in case it happens, I'm I'm very scared now of driving by myself because I have been in the car when it's just come on all of a sudden and then I've been like mad full trying to get home in time, you know. And I mean, I've learned now myself kind of how to control it myself |
| | | | about three Times Now they've said, you know, if you if you had left it another hour, this would have gotten to sepsis and, you know, we would be fighting for your life. And I think that's made me really scared |
| | | | I've been told it's a life threatening illness. And if I don't get to hospital in time, you know it it it could be really serious |
| | | | I've feared that the antibiotics could do terrible things to me, my organs and stuff |
| | | | I think the fear is that for me, I feel I won't live to be an old age, but I've accepted that |
| | | | I fear how long you know this is gonna help, these at these tablets are gonna last on me for |
| | | | I would say my fear is the long term effect and even though it is a bit gloomy, I do want knowledge on how it could affect me long term |
| | | F-58 | It's been from being a confident, happy, positive person. You know, I used to stand in front of 200 people and give a talk. I'm so scared of going out. Because not because I need a toilet, but because I'm in pain and it's pain that makes you fearful |
| | | | My overall being overall well-being is absolutely shocking. I have counselling once a week. I watch online videos. I do meditation. I try so hard. But it's it's just appalling. Every single day I wake up, I think ohh God, here we go again. You know what have I got to get through today? I'm constantly stressed. I'm constantly in this fight and flight mode, as I said. And I try not to panic. I try not to whirl off into as this forever. Am I ever gonna get rid of this? But it's very hard not to have those thoughts |
| | | | I can't begin to describe how debilitating this is. I had a lovely job, you know, I've worked a major public school for 23 years. I was in and out of, you know, boarding houses. I loved it. I'm a very sociable person. It it, it was just fantastic, you know? And I used to go to the theater a lot to the cinema and just I'd always be the first one to go and rush off and do something and then exhibition or whatever it was. And you know, like travelling etcetera. The carpet has been pulled completely from under my feet. You know I'm. I'm practically home based |
| | | | the most difficult thing about all of this is my relationship with my husband because. You know, it would be married nearly 30 years and he's he's a good man and he's so he's, you know, he really does his best, but he doesn't know what to say anymore. And I feel, you know, my life has shrunk and his life has shrunk as well. And I'm I'm. I'm tearful, I'm unhappy I'm I don't want to be needy but I am and you know the whole balance of our relationship has shifted because I was always the one who was in control and organizing things organising social life |
| | | | My daughter's gonna get engaged soon and that you know, it should be a really happy time. But. [participant is tearful] I don't know if I'm going to be able to manage it. You know? How do I go and look for a dress with her? |
| | | | I think I always worry from infections. I mean not just from my peg site but also from other groups |
| | | | This is gonna damage a little bit. Yeah, it's it's living without fear and that's all in your head and you don't worry about the future because when you hold, |

| | | | |
|---|---|---|---|
| | | F-52 | everyone naturally gets more tired, gets more, you know rundown like for hours. Well, I already feel like I'm waiting results and bodies, you know. And it's. You do get worried about future infections. Yeah, especially how quick an infection can take over |
| | | | you do worry about it. You know you're always trusting or anxiety about sit and you know. |
| | | F-61 | it's a very, very scary place to live, never knowing and never knowing from one day to the next if you're going to be well |
| | | | I think probably the the thing that is. It's the scariest is to imagine that maybe one day they'll have to remove my bladder and that really freaks me out. Uhm, that's what keeps me going. Actually keeps me fighting |
| | | | now my worst fears have been realized with my husband. And and I don't know the I don't know how long he is going to have, but. And I have been told. Not to look into the. Future but I. Am going to be widowed. And I then think my my big protector, my my lovely husband who's looked? After me, for all of these. Years, who's going to look after me? |
| | | | It seems to evolve and it becomes resistant to that and then the doctor says, well, you've only got 2 left and. You think, Oh my? God well, am I going to die? And it's really scary, really scary. And so. Yeah, I I do. I live in fear. You be honest. I live in. Fear all the time |
| | | | So what happened is that has become just. Extremely resistant over this. This two year period and there's that doesn't seem to be an answer for me at the moment |
| | | | I had to manage my my mind with the infections and I'm not going to lie. You know there's been times. And I've honestly felt that I couldn't cope anymore and that. I mean, I'm I don't think I ever would have committed suicide, but I have felt suicidal at times. Just thinking I'm a rat in a trap. I cannot get out of this cycle |
| | | | the situation I find myself in now with my husband as well as he is I I don't have another option. I have to be well and and so it it is making it is adding to my stress in an extremely acute way and. Yeah, I would call it intolerable |
| | | | I'm just not winning. And it is actually. Ruining my life completely and at the moment it's making it utterly intolerable |
| | | F-48(2) | and I'm really independent into trying to get all his stuff as much as I can, you know, but it gets. I cannot drive my car and I've got an automatic. Adaptive car and it's really quite frightening |
| | | | I'm terrified of losing my independence. Absolutely terrified of losing my independence. And I just don't, you know, I don't know what happens. Uhm, and if I couldn't advocate for myself, I've got absolutely no confidence because it seems to be quite complex and a lot the health professional don't seem to understand it at all |
| | | | I'm really cautious because I'm so frightened of getting any another sort of an infection that I that won't. You know they won't. Uhm, be treated |
| | | M-78 | I lost my appetite. That was probably half due to the antibiotics and due to the stress caused by the fact that I might […] lose my foot on my leg. So that was a massive […] Was all a big part of the stress on me. |
| | | | even though I'm not one to get stressed out […] and so I didn't need attention for the mental, the mental illness, if you like. But it was it was having an effect in a certain way; appetite or being very snappy to people, things like that, which again is something that they recognise, that you do, that you do get irritable with these symptoms, you know. |

| | | | |
|---|---|---|---|
| | | | mentally and obviously on my, like just everyday way of life, you know, changed. Because of the constant worry. Just a constant low for several months when the doctor told me |
| | | M-26 | Yeah, I worry because like now the microbe resistance makes you really dependent on medication and that's something that I really had to agree with being dependent on, having to use to use drugs, and it's really challenging. |
| | | | Yeah, it has affected I think I would say a lot of my confidence cause, more like an adult person and not an adult, you know, I mean, because of that infection, I can't imagine going to the swimming pool or going out because it's affected a lot of the confidence that I have in my body. |
| | | | It's affected more on my mental health. Yeah. The, the effect I feel more mentally. |
| | | | Because I feel that at times I just get lost on thoughts about the infection and I wonder if I would ever be free from this infection. So like yeah, I worry about it. |
| | | | Well, I just, it's really taken a lot of a toll on my mental health |
| | | | I think that it's important that a lot of people know about this condition early and you know giving, teaching information about the condition early on. That's really important, and the mental support for people who are going through this. You know, if that can all come through the NHS you know if you can talk to someone about having this condition, mental support, that would be fine. |
| | Feelings of Shame | F-40 | I felt like I just came out worse off, you know (.) and you get no apology I got no apology from the hospital whatsoever about what happened when they wrote back to me they just tried? They actually. They blamed it on myself, they said I gave myself the infection through my own number two, my own excrement |
| | | F-48(1) | There wasn't much information given to me while I was in intensive care and then they just put me in a room on my own and. Said that wasn't allowed out of the room. No, it wasn't discussed, I had to come home when I was well enough to find out everything myself which was difficult |
| | | | I had a tough time 'cause when I went to A&E I wasn't treated very well and 'cause I'd gone there very shivvery and really poorly and I was asked and they wanted to do an HIV test and doing swaps vaginas swaps to see if I had sexually transmitted disease any infections sorry and I was saying to them, you know I'm a happily married woman and I was so upset and angry . You know about the way that I was treated, and you know, I was left on this in this room on my own |
| | | | they would not let me out of that room and nobody came in and spoke to me about any of that, it was awful |
| | | | I was going to the bathroom and there were two nurses talking and they said she's an infection risk. So I said he talking about me, she went no. So I came out of the bathroom, sat on my bed and they came up to me and said, we're moving you, I said, well, why did you just lie to me? I said you was talking about me there in front of all these patients I said, you know you should, why didn't you come up to me and speak to me privately. So now everybody else is going to be worried that I've got some kind of disease and big infection that they're gonna catch, because they're all sharing the same toilet, so I was very upset over that |
| | | | they had no right to speak about my business in front of all the other patients. I said I'm not embarrassed over it. But you know, it's patient, patient confidentiality and I've said they've just lost my trust now |
| | | | you've got these super bug 'cause they're gonna be all worried and if nurses can't explain to the rest of the patients that they're not at risk, who's gonna, me? Go round to everybody and say look, you know you might have heard this, but you're not at risk, that's for them to deal with |

| | | | |
|---|---|---|---|
| | | | I'm thinking, you know, I'm just left just a patient, I'm a nobody, But why don't people come and speak to me about it and I just felt like a lepper I'm in this room and got all this stuff on and. Oh, you've got to say that you're not allowed out of this room. We're going to come in and. Do special cleaning your toilet and shower |
| | | | and they should all gowned up and everything and I said why have you got all that on what's going on? You know, and she goes, oh, you've got an infection, you know, we've got to be careful and she said you're not allowed out of the |
| | | | then I thought you know, how can I go home? Am I going to pass it on to my husband, my children? There's just no information because it sticks in your head when they're saying you can't go out of this room and they're all gowned up. How can I go home and you know, am I going to give it to my family, my husband, my friends. There was just nothing at all. You hear about the MRSA, get swabbed for that, but nobody does anything about the ESBL |
| | | F-61 | I know GP says they've got a difficult job to do. Uhm, but because I am a a frequent. Unfortunately, UTI sufferers you you tend to find that dumb. They almost it's kind of I owe you again and and and you start getting lectures on how many antibiotics you're taking and you've got to bring your antibiotic consumption down. As if I'm doing something wrong and and the latest. Kind of. Approach from GPS is try to leave your symptoms for as long as possible so that your your good bugs can try to get on with fighting |
| | | | You are made to feel. A bit of a nuisance. And and there's an expression that one of the doctors used. At one point |
| | | | can't be the only one going through this, but yeah, dignity, humiliation. Made to feel. Just that you're not actually that. It doesn't seem to matter, you know? Just take the pills and go away |
| | | F-48(2) | I did ask the specialist nurses, you know. Is it something I'm doing? You know, is it something I'm eating? Is it something I'm T touching? Is it kind of my pet, you know, is it and they were like no, it's just it's in you |
| | | | even my mum. You know my parents are just like. Well, you're or. You've always got an infection. You must be doing something wrong and I'm like. If anybody can see what my daily existence is life to like to try and just stay well |
| | | M-26 | I think it's affected my relationship with my close friends. Since they found out about the infection, you know, its more like they're very careful around me because they're afraid about the contagious, you know this feeling of not being very comfortable like I used to be with my friends. If I'm round someone's house, I see that they could be just watching my every move. You know, it's just… |
| | | | I feel I would prefer that support coming through private because I wouldn't feel comfortable talking about this condition with other people around. I feel I would like that support to be more personal. |
| | | | Researcher:<br>Yeah. So would you feel like a kind of patient support group, you know, in a group setting would? That wouldn't be so helpful.<br>Participant:<br>Yeah, I don't want that, I feel like even if we had the same condition, you know, I wouldn't want that. |
| | | | I think a lot of people with my condition would actually need more personalised support. Yeah, more personalised support. Well, you know, a lot of people actually, they call having this condition it's very private. I think it has, if somebody were to ask me, you know, I'm just thinking it's going to be private for a lot of people. |

| | | | |
|---|---|---|---|
| | Feeling Infested by AMR | F-40 | Then for some reason around about 5:00 o'clock, that is when all the (inaudible), you know, those 'cause. It's believed I have a biofilm infection. I feel they just want to come out and play and cause havoc |
| | | | whilst the infection was just taking hold of my bladder and, you know, creating biofilms, as I understand it now. Uhm, you know, and creating homes in my blood. You know, creating little homes and it was just what I think they're doing. So they've got friends, you know that live inside me |
| | | | I'm open to sepsis now, but I'm obviously, the antibiotic is controlling, you know, the infection in such a way that hopefully that it's keeping it at bay and avoiding it |
| | | | I also described mine as persistent as well because I I don't think it's ever left me, I've never had a downtime if you know what I mean? I've had time of when I feel I've got no flare ups, no symptoms |
| | | | These things that live in me, they tend to say we're coming out to play at 5:00 o'clock to cause you pain and havoc and to ruin your life, you know |
| | | F-58 | these are quite serious bugs and I just can't get rid of them |
| | | | what's happened is that the bugs have been stunned. But they've delved deeper and deeper into the bladder wall. I now know this. They've been allowed to colonize. They've been allowed to build biofilms. And that's why I'm in this mess |
| | | F-48(1) | then I thought you know, how can I go home? Am I going to pass it on to my husband, my children? There's just no information because it sticks in your head when they're saying you can't go out of this room and they're all gowned up. How can I go home and you know, am I going to give it to my family, my husband, my friends. There was just nothing at all. You hear about the MRSA, get swabbed for that, but nobody does anything about the ESBL |
| | | | I was scared to come around and you know, using bleach on the toilets constantly. My hands were going really sore because I would just constantly putting bleach and I used to say kids, because we've got 2 bathrooms, 'cause I've got my own, and I just said please don't got my toilet, don't go on my toilet I were constantly washing my hands. You know, and I I was so scared to give it to them and then once I've got more information and I realized you know that they're not at risk just, you know, good hygiene, which I was doing anyway |
| | | F-61 | my consultant tells it that I've been colonized because I've had this for so long |
| | | | I've had to cancel so many things and I hate canceling things. I hate being that person. You know, I want to be a robust helper for my husband. I want to. Be a reliable friend. I want to be. A reliable, fun Granny. There's lots of things. I want to be. But this stops me in my tracks over and over and over again. It's not allowing. Me to be. Who I was |
| | | | It's made me really seriously depressed at the thought that, yeah, I'm just not a reliable friend. I'm not reliable. Mother wife. Uh, you know, I'm I'm I'm not in control of my body. Something else is in control of my body. And that's what it feels like |
| | | | gradually, gradually, as I say, they went from one a year to one a month and now it's one a week. And because the antibiotics are just, they're just not killing the bugs. And I understand that what happens is they kind of burrow into the bladder wall set up camp and particularly oral antibiotics, just never really get to them |
| | | M-26 | Yeah, it has affected I think I would say a lot of my confidence cause, more like an adult person and not an adult, you know, I mean, because of that infection, I can't imagine going to the swimming pool or going out because it's affected a lot of the confidence that I have in my body. |

| | | | |
|---|---|---|---|
| | Feeling Isolated and Not Understood | F-40 | These things that live in me, they tend to say we're coming out to play at 5:00 o'clock to cause you pain and havoc and to ruin your life, you know |
| | | | all of these things are invisible. No one can see any of this. You know, it's like I'm not walking down the street with a sign on my head or or, you know, or a visible disability where people open the door for you or understand you need a seat on the bus 'cause you're feeling dizzy or whatever |
| | | | no one understands really until you explain everything to a person how it feels to live with invisible illness |
| | | | My partner did say some of my personality changes and she I'm I'm with the lady and she can see it in my face that I'm in such pain that you know, it's like my face contorts or something 'cause, I can't, I can't express it. It's just so painful. Uhm, I find that my mother that didn't really understand it. She understood the stroke more I think as my grandmother was susceptible to strokes, but different kind than mine, but no, people don't really get it 'cause you don't really look unwell, that's the problem |
| | | | I think it's just like if I told them anything, it was like, oh gosh, that's terrible [laughs] but I don't think any real true understanding really. I've only found really true understanding from people that are going through the same thing, and they've all found the same problem with telling friends and family that because they they look alright |
| | | | it's why I've cut down considerably on my personal, you know, life, socializing is because I get flare up so often every other day, every day. When it when it comes to certain time, 5:00 o'clock I know I'm that's it, I'm done. And so I often if I do something and I have a day off, I try and do it during the day |
| | | | I remember just sit sitting there wishing the time I'm away and it's horrible 'cause I went there with my partner who deserves equally a good time, who's there for me in every way you know, possible, I'm so grateful. And I feel like I just ruined things, you know? Because I'm there miserable and trying to be OK, which is really fake |
| | | | That's what I've understood from my situation. You've got to look unwell for anyone to understand your problem |
| | | F-50 | I got my infection and even though I was controlling it still felt really rough and I had to cancel it and I just feel really guilty when I have to cancel on people and I really hate it…. But I don't wanna be an inconvenience to them so I just say you just go without me |
| | | | whereas my mum and you know, she's she's an older generation so she's found it a lot more difficult to understand, I think. And. And some of my friends just think or, you know, isn't infection. I'm on tablets for it. So everything is fine |
| | | | they see me dressed, makeup on, they think I'm fine. They don't really know what's going on inside |
| | | | a majority of them don't even ask questions, you know they see all these tablets I've gotta take uhm. Last time I was away with him, one of them did say well explain to me what all these tablets are for, and I thought, well, that's really kind of her even if she's really not interested that's really kind of her to ask, whereas the others haven't got a clue what goes on |
| | | | I have to cancel so many things. And I said I can't invite people over unless they can pop round for an hour or so. I can't create. I can't have a meal or something. And you know, I just think to myself, will I have any friends left, you know? So I'm a very, very sociable person. I've got lots of really good friends, but I don't know what can they say to me anymore, you know, and I and I find it very |

| | | | |
|---|---|---|---|
| | | F-58 | difficult when people talk about all these exciting things they've been doing and holidays have been going on. I I find that really hard . |
| | | | my husband, you know, we can't have any sexual relations that's just gone completely out of the window. And as I said, I I don't want to be this needy person, you know? And and sometimes he just has to go out. He just has to leave the house for a couple of hours a night. I totally understand that. But that makes me feel even more guilty |
| | | | you do slip back into your relationships and that's all that keeps me going just to think, one day I'll get rid of this or I'll be able to live life comfortably, and then maybe I can repay the kindness that the friends have showed me and I can be the friend I want to be to them that I that I used to be… they say. Oh, gosh, that's awful. But you look fine. Yes, I look perfectly OK. But you know, I'm. I'm really suffering |
| | | F-52 | You know it's hard enough for like normal conditions, everyday without having an infection on top of that, you know, like sometimes I don't feel well and have to cancel plans and spend another day at home |
| | | | my friends, not that I can make plans with swimming tomorrow, but then I might have to bring up tomorrow and say after that comment. I'm not doing one today and we get it and you understand and we certainly understand |
| | | F-61 | I've had to cancel so many things and I hate canceling things. I hate being that person. You know, I want to be a robust helper for my husband. I want to. Be a reliable friend. I want to be. A reliable, fun Granny. There's lots of things. I want to be. But this stops me in my tracks over and over and over again. It's not allowing. Me to be. Who I was |
| | | | It's made me really seriously depressed at the thought that, yeah, I'm just not a reliable friend. I'm not reliable. Mother wife. Uh, you know, I'm I'm I'm not in control of my body. Something else is in control of my body. And that's what it feels like |
| | | F-48(2) | it affects every aspect of your life. My partner left me because they were so kind of thing about I was just always ill and unwell and couldn't understand why nobody was like, was helping me. Yeah, uhm and just find it too much to watch. Just found it too much to watch somebody just being so, you know, so unwell |
| | | | it's just exhausting, because it never goes away. And people don't understand. Like people don't understand, they kind of think Oh well, if you've got an infection, you have some antibiotics and that's it |
| | | | even my mum. You know my parents are just like. Well, you're or. You've always got an infection. You must be doing something wrong and I'm like. If anybody can see what my daily existence is life to like to try and just stay well |
| | | | I'll get a load of suggestions about how it can just be sorted out and there doesn't appear to be a solution at the moment. And the last thing I need is another load of people telling me things that like all gonna make a blind bit of difference |
| | | M-26 | Well, I see that people are really less supportive. That's, I think, that people are really less about this, about my condition. |
| | | F-50 | I was rushed into hospital and they put me on antibiotics and I had a lot of tests after that and and then it was, I was told that I had this in infection. The, I would have to come back in hospital basically on and off for the rest of my life, yes it was something I would have to live with. And so it was a bit of a shock 'cause I didn't know anything really about infections |
| | | | I can't begin to describe how debilitating this is. I had a lovely job, you know, I've worked a major public school for 23 years. I was in and out of, you know, |

| | | | |
|---|---|---|---|
| | Feelings of Shock | F-58 | boarding houses. I loved it. I'm a very sociable person. It it, it was just fantastic, you know? And I used to go to the theater a lot to the cinema and just I'd always be the first one to go and rush off and do something and then exhibition or whatever it was. And you know, like travelling etcetera. The carpet has been pulled completely from under my feet. You know I'm. I'm practically home based |
| | | F-48(2) | Thing is, and I never took antibiotics as a child. You know, I'm not somebody who's run back and forth to the doctors and all the rest of it. I got hit from nowhere with them |
| | Sentiments of Hope | F-50 | I'm eight years in now, and they're still working and I have learned that, you know, these tablets can tragically be resistant to me, but you know there is hope, that there is another one or maybe two out there that can help me and to never give up hope because they are working on making new antibiotics all the time |
| | | F-58 | you do slip back into your relationships and that's all that keeps me going just to think, one day I'll get rid of this or I'll be able to live life comfortably, and then maybe I can repay the kindness that the friends have showed me and I can be the friend I want to be to them that I that I used to be |
| | | F-61 | I live in hope that somebody will. Somebody will come up with an answer, but until then. All I've got is this. Crazy life of begging. Basically begging. To be not even well but made cut into a position of being able to function in my life |
| | | F-48(2) | it's probably made me stronger because I try and focus on the positives. Like as in. Just think about the things that are positive and. Because I can't, it will just overwhelm me if I actually think if I actually sort. Of listed that. Impact they that it has had and has had |
| | | | sometimes they do. It does work for a little bit and I'm like Oh my my life is like transformed and I. And I think this is how I can feel and this is what I say to the doctors. Quite a lot or to the the specialist here. I know I can feel much better than I am physically. I know we can and it is such a relief when that happens |
| | Symptoms and Side Effects | F-40 | it's really difficult really because you try and enjoy yourself and you can't because quite often I have I have like days of feeling nothing like I have no infection and I'm really grateful for that. And then I have other days where I 'cause, I've now developed incontinence from, from the infection as it progresses, the disease progresses |
| | | | I was getting covered in there and infection on my. She's just bizarre, isn't it? Uhm, wells Pip miss selenium [Piperacillin]? I think that's how you say it, I took that during the first lockdown, and I ended up in bed for weeks on end not knowing it was the antibiotic side effect. Um, it made me really depressed. I was tearful. I couldn't. I was sleeping 20 hours a day |
| | | | Every, every day, every day for me now, I was saying they last erm I actually would say over the last year, I think I there's very rare occasion I have two or three days without a flare and I do get them, but they're so they're so kind of, like, rare don't really record them if you know, I mean, but no I do get them a few days sometimes. Around my period they can be worse. Really violent, really painful. Um… Yeah, they can be very, very… and the incontinence can be can be worse as well |
| | | F-50 | Living with the infection, it is the uh, I'll get a bad taste in my mouth, which is really drives you crazy but. You know, I drink a lot I try and drink a lot. And and I, you know, probably get through far too many sweets [laughs]. But you know, that sort of things effects me the grogginess of it all, other personal things that antibiotics can cause for ladies and thrush and things like that I have to deal with that on a on a daily basis |

| | | | |
|---|---|---|---|
| | | | I've been on them now for it's coming up to 8 years. And things have started to unfold a little bit more. Now I'm getting more problems, more infections |
| | | F-58 | I've had long term high dose antibiotics. I was getting a lot of side effects. I've tried every alternative treatment going and I've still got it and it's very, very debilitating and you know, every day is very uncomfortable and it's mentally very, very challenging as well |
| | | | my biggest problem is, as I said, side effects with antibiotics oral antibiotics. I had terrible bacterial vaginosis and thrush. I mean you you can deal with that |
| | | | So I've got huge gut problems and I've also been getting peripheral neuropathic pain. So. So I've got sort of singing up and down my legs. It feels like I've been plugged into the National Grid and that started last summer when I was on amoxicillin and now every single antibiotic tablet I take causes the same really scary neuropathic pain |
| | | F-52 | It hurts. You can feel it in your stomach where it is and it is very bad when changing dressing, you have to be very gentle. Mean sometimes like when my nephews comes and hugs me… yeah, kind of hurts when they try to hug there. |
| | | F-61 | I've definitely gained weight and I notice that when I tried to climb a hill, right? Particularly if I'm on antibiotics get very breathless. I'm much less fit than than I was |
| | | | Sorry for all the details, but this is real and if I'm having it expecting other people. So you know you end up. Using, having having to use batteries for the piles and then continue to take like stiff 'cause you're still constipated and you know trying to eat fruits and vegetables and fiber and doing all the right things. But you're in this hideous, vicious circle |
| | | | I'm supposed to have a cystoscopy on the 13th of August and and I woke up on the 13th of August. It was this Saturday. And I had to cancel this discussed cystoscopy because I had a bladder infection |
| | | M-78 | I lost my appetite. That was probably half due to the antibiotics and due to the stress caused by the fact that I might […] lose my foot on my leg. So that was a massive […] Was all a big part of the stress on me. |
| | | | even though I'm not one to get stressed out […] and so I didn't need attention for the mental, the mental illness, if you like. But it was it was having an effect in a certain way; appetite or being very snappy to people, things like that, which again is something that they recognise, that you do, that you do get irritable with these symptoms, you know. |
| | | | as I say it makes you a bit irritable at times. […] I am aware of it, and I know that I lost my appetite because of it. |
| **(Theme 2)** I'm battling on my own: A journey toward self-stewardship | Little Knowledge of AMR Among Clinicians | F-40 | no one can help. No one knows. No one knows what to do. No one knows what it's like. I haven't found anyone else who is in the same boat as me and not even my GP knows what to do. 'cause they just don't know |
| | | | When I went to my, I think 'cause I was seeing a student who I thought was fantastic, better than the experience Doctor to be honest, they both sat there saying we've got no idea what post Sepsis syndrome is. And I said well it's on the NHS website [laughs]. But I thought that's really interesting, you know? And they both sat there looking embarrassed and puzzled at the same time, they don't know anything about the, you know, antibiotic resistant infections and not even the pharmacist that issues the, um, the medication for me at the doctors surgery |
| | | | she will order a, I think it was a test, the urine test and to see if I have an infection. And I thought, well, I have an infection. Well, there you get all the information from the doctor. I see it never goes. Um and, you know, just 'cause you want to avoid resistance. And I thought, but I've already got resistance. And |

| | | | |
|---|---|---|---|
| | | | I thought it's amazing that people in the medical profession are still not in the know-how of what is going on. You know, and I can't come off antibiotics. I've explained that before because it's dangerous for me to do that |
| | | | I now have private health care in place just in case I have to go through something. But no one knows anything, the information, they're not as educators as they should be. I know there's nice guidelines out there. I've read most of them. They're supposed to give you information about sepsis and post sepsis syndrome and and what happens to you after sepsis. None of that happened when I was in hospital |
| | | | there's not enough knowledge out there in with the you know, in the NHS and their GP and the the clinicians and the surgeons and [laughs] |
| | | | not even GP's know that information they don't really understand that as well 'cause I've tried to speak to them, they just don't know |
| | | F-50 | And they they don't really know. I'm teaching them something, so it is a lot of it is a lack of knowledge and communication and if there was more of that, I think in the earlier days I would have been more aware of what was happening to my body |
| | | | I've tried asking all these questions to my consultant and they just they said that there's so many bacterias that is involved in it that they can't point it down to anything… I've tried asking questions, and it's as if no one knows. You know, it's like they just don't know. So I've not ever got the answers I've wanted |
| | | | he didn't really know himself, to be honest. And he he used to call me his golden ticket because they used to. I think, you know, doctors are restricted for how many antibiotics they can give out to patients |
| | | | It would be nice if my GP understood it more. You know that side of things it is at the end of the day, it is lack of communication and it isn't that lack of knowledge. I mean, I'm learning about it every day. Uh, you know and a GP obviously is just a General practitioner |
| | | | I think they definitely need to know how antibiotics work in your body. You know how they fight the infection, how long term it can affect you, the side effects. I think they should really be aware of that side of things |
| | | F-58 | The healthcare professionals are not equipped. They're not equipped to deal with these infections adequately |
| | | | I was speaking to my doctor just last week. It's beyond their remit. They don't know what to say. They've done know what to do with me and you know, dare I say it most urologists don't have a clue what to do either… So, you know, I've got to the stage where I think you know, what's the point, because they really can't help me. They can sympathize, but they can't do much to help me get rid of this |
| | | F-48(2) | I'm terrified of losing my independence. Absolutely terrified of losing my independence. And I just don't, you know, I don't know what happens. Uhm, and if I couldn't advocate for myself, I've got absolutely no confidence because it seems to be quite complex and a lot the health professional don't seem to understand it at all |
| | | | I don't think it's like they've got a load of information. That they're not giving me. I think they don't have it. |
| | | | I really I don't understand and I've tried to add some unit. Does that mean that I'm antibiotic resistant? Everything, So what about if I get another sort of infection or or, you know, God forbid I had some sort of an accident or something like that and I needed some treatment. Does that mean I can have antibiotics and nobody seems to like know or answer |

| | | | |
|---|---|---|---|
| | | | they gave me 100. Too many it would have killed. Yeah, they just don't know what they're doing |
| | | | they've just been like there aren't any options. There are no options and we don't know what to do, so. |
| | | | I know because of my test results, so I went and asked the GPS. Haven't got a clue like literally. Don't have a clue |
| | | M-78 | Information I've read and taken to the various doctors and consultants that I've seen and told them about it, you know, because they're just not aware. |
| | | | The same as doctors, they're not aware with warfarin, they're not. They're just not aware of the different aspects of it. I had to ask my doctor to phone up the biologists at the hospital to ask them which he very rarely will, the doctor do something like that, and they think that it's necessary and you pick up the phone and do it straight away, and found out that it was gramme, negative bacteria actually. So, this is the sort of things that the doctors don't know because they're general practitioners, they're not specialists. |
| | | | only the specialist, the vascular surgeon, will be aware of all these different parts you know. As I say, when I saw the specialist because when I saw that I went to the A&E and I only saw the orthopaedic surgeon, and we don't have a vascular surgeon resident at my local hospital. He only visits that's all the once a month or something, you know, he holds the clinic say once a month. So that's why there wasn't one there to see that. […] And again, they're not familiar and all they did was to send off pictures of the ulcers in the post; there was nothing done at all to check for the osteomyelitis. And the ulcers in the toes can obviously get worse […] so that, that that was one big concern of mine |
| | | M-26 | I think finding the professionals with the knowledge about this condition could be a barrier |
| | Lack of Care and Support by Clinicians | F-40 | She did everything she could within the guidelines, but they they their hands are tied, and I think that's where the issues start as well. And the avenues I had explore to try and get rid of this infection not knowing this has become resistant I mean why was it down to me to do all that? To contact the Secretary of the surgeon to get into the cancer clinic to contact Doctor Ron Daniels to get a letter written to say, maybe try this antibiotic and him knowing that there's guidelines |
| | | | Luckily my surgery are so and I think it's 'cause it happened within their hospital trust, why they're paying for my medication, although I'm getting the help privately they're paying, which is very unusual 'cause even Professor [M] said they don't usually find that GPS will allow you to have it on the NHS. Usually, you have to pay for it privately, which I haven't got the money to do that. I mean, I think if I was made to do that, I I don't know how I'd afford it |
| | | | I have investigated phage therapy and I'm very interested in doing it, but I haven't got £20,000, the cash, for treatment that was actually a treatment before penicillin even came in. You know, I mean, I find that bizarre that I can't access that kind of help. |
| | | | like other sufferers, as I found out, I've spent hundreds of pounds on biofilm Busters, you know, supplements that can penetrate the biofilm and get through and try and kill it and nothing happens. Nothing happens, I'm afraid, so I've tried |
| | | | So while I am looking towards uh controversial treatment or a miracle to happen or I don't know, I don't know. Or one day I can afford to go and try phage therapy or do something different |
| | | F-50 | I was in and out of hospital all the time (.) but I wasn't really told how the antibiotics work. I wasn't told anything about resistance, I wasn't told anything really |

| | | | |
|---|---|---|---|
| | | | when I had my appointment, it was basically how have you been at the you know, how you taking the tablets, blah blah blah. And you know, if you go sort of, see you in another six weeks. Uh, and I tried asking questions, and all they would say to me was well, we've put you on three different lots of antibiotics, so this will slow down the resistance of it all and and while it's all working, everything is fine. So I never really got a chance to find out what I wanted to know |
| | | | I left that hospital with no information about, you know, if my scar, well uh, gets an infection or anything like that |
| | | | I really wasn't educated well in it at all. And I knew antibiotics wasn't good to be taking because of resistance, but I just thought well, you know, I've been put on these by these consultants, so it must be OK, you know, I mean I'm just was very poorly educated in it where they |
| | | | he did say, you know, it could affect your stomach and your bowel, you know it, it could affect other organs. And he said he did say, you know, and it could be that once your body is resistant to these antibiotics, then we'll just see what other antibiotics you can take. And it just made it seem like, oh, fair enough. But it wasn't until I spoke to my consultant a couple of months after, maybe a year after that, he said to me with my infection, there's only five antibiotics that will work for it, and I'm already on 3. Uh, I started taking one right at the beginning that had really bad effect on me, so I had to stop taking that one. So at the moment there's only one over the I could use, so you know it's not, it's not very good at all |
| | | | I think I'm consultants and doctors like explain things to in medical terms that you know. I always say, can you just like, tell me in plain English, you know, 'cause, I don't understand these long medical terms. So, you know, I think for me I'd rather just be told in black and white what it is I've got and what's going to happen |
| | | | I understand that the health professionals are extremely busy but I I can honestly say I've not really had any much information from them. It would have been very nice if I had. It would have made my life a lot easier at the beginning |
| | | | So I just thought it would one course of antibiotics, and then they explained to me after I think it was about a couple of weeks, I had to go back at six weeks and then they told me they were gonna be putting me on three different lots of antibiotics on a Rotary course |
| | | F-58 | I speak to other women in the same situation, which gives a bit of comfort, but the conversations with the health care professionals I've found to be pretty useless |
| | | | I'll be honest with you, the every single test I've had done on the NHS has come back negative and I know full well that I have an infection |
| | | | I went two urologist who basically said to me, look you haven't had an infection all these months. This was about September. I said well, you know I'm the patient. I'm telling you, I don't feel right even though you tell me I on paper I haven't gotten infection. So that's how it all started and I've been battling the same thing ever since. |
| | | | your urologist, you'd hope would be able to, you know, advise or give you treatments that that would actually help you rather than barbaric bladder stretches and cystoscopies and goodness knows what they do. Just because I think they got to do something, it it can make people so much worse. Luckily I haven't experienced any of those myself, but I only know that from the from the |

| | | | |
|---|---|---|---|
| | | | Facebook group. People say don't have this done, don't go and do this. So no, I haven't had sufficient information. Absolutely not |
| | | | the specialist I was under in Harley Street doesn't actually check, doesn't do a broth culture, so just treats on symptoms |
| | | | when you're diagnosed, what would you? You're accepted as having an infection even if it doesn't show up on the test. We're giving three days' worth of antibiotics. Well, they give cats more than that if they've got an infection |
| | | | if somebody says they've got a UTI will say go to the doctor and insist on getting at least 10 days, two weeks worth of antibiotics. But you they'll be lucky if they get it because they're their hands are tied , because that's not the protocol |
| | | | I've seen a couple of people privately, as I said, who are prepared to sort of stick their neck out and try something that's not run as a mill. They're very few and far between and it's very expensive. You know, you have to pay an awful lot of money for this. And I'm not working. I can't work. And there's so many people in the same boat as me. How can you work |
| | | | we need new treatments and we need money ploughed into new treatments now |
| | | | Something needs to happen. The initial testing needs to be changed. The treatment options need to be improved, that there are new treatments on the horizon for my particular condition, but they can't get the funding. What more do we have to do to get funding for these infections? |
| | | F-48(1) | There wasn't much information given to me while I was in intensive care and then they just put me in a room on my own and. Said that wasn't allowed out of the room. No, it wasn't discussed, I had to come home when I was well enough to find out everything myself which was difficult |
| | | | There's just no information, and then you discharged you go home and you just left with it, you know. And like I said, I've had to do my own research and calling and trying to find out about ESBL |
| | | | As you know there the caregivers are supposed to have all this knowledge and come and speak to patients like if they're diagnosed with cancer or something. You know they'll come and. You'll go in for an appointment and we discuss it face to face. Why couldn't they have done that with me with ESBL |
| | | | A doctor came in and said, you know what's been wrong with you and I said I had sepsis and he said you didn't just have sepsis you had septecemia. I said oh OK, I thought they were same. And then I said well what about the other thing, ohh that's fine, you're OK, and I never got any information |
| | | | I'm thinking more you know, what have I got what, what's going on, and then you're getting told oh you're OK. It's not good enough . Very very poor |
| | | F-61 | I know GP says they've got a difficult job to do. Uhm, but because I am a a frequent. Unfortunately, UTI sufferers you you tend to find that dumb. They almost it's kind of I owe you again and and and you start getting lectures on how many antibiotics you're taking and you've got to bring your antibiotic consumption down. As if I'm doing something wrong and and the latest. Kind of. Approach from GPS is try to leave your symptoms for as long as possible so that your your good bugs can try to get on with fighting |
| | | | if I do need intermittent oral antibiotics, I have to go. I have to. Go through the whole GP. Up some battle through the receptionist, get speak to a doctor. Gets more antibiotics and this is all just to stay functioning. And you know, I don't really feel I've been given any information or really much help. I'm I'm on my own battling alone |

| | | | |
|---|---|---|---|
| | | | A sense of being cared for would be more help and a sense of continuity of care would be more help. Uhm, because Honestly, I feel that with every episode whenever infection episode, I have to start at the beginning and I have to work like the devil to get through to the doctor's surgery to try to have a conversation with the doctor and to try to get antibiotics in time and and it's. Such an effort when you're not feeling well |
| | | | I always feel that. Mostly, I'm almost asking for something I shouldn't be asking for, particularly in recent years, despite the fact that I am clinically unwell and I've had a positive pressure almost every time. You have to convince them that you're you're ill enough |
| | | | I always have to ask for the standby antibiotics. So as I say there's there's never a case of. How can we make this easier for you? It's always me having to do. All the push all the hard work |
| | | | You are made to feel. A bit of a nuisance. And and there's an expression that one of the doctors used. At one point |
| | | F-48(2) | I might sound a bit confusing, but the situation is quite confusing because I'm between two hospital trusts and also. The services don't match up. There are loads of different people involved in my care in different places |
| | | | it's very disjointed, so there's a very low level across most of health care about understanding this. And you have to constantly repeat or explain or take. So I take if I end up places or take a list with me or a print out or something |
| | | M-26 | And then I think the cost of actually accessing the support. |
| | Feeling Dismissed by Clinicians | F-40 | I go and see the surgeon. Uh, during his cancer unit that's um, sorry clinic. That's the only way they could get me in to see this guy. UM, no apology, no nothing. Nope. Absolutely. So strange |
| | | | when I went to pick up my medication the other day from the a different pharmacy that I don't use, the lady looked at the amount of antibiotics that I had to pick up and she said to me, what's wrong? She said [laughs] there's so much here, what's going on |
| | | | Oh my experience with NHS professionals. I felt like just wasn't listening to actually, uh, you know, the ambulance crew, I don't know what happened really |
| | | | when I said I was in pain with you know the urination and so on. I was literally dismissed. I read my hospital notes. The last doctor I saw two hours before I was discharged, I I told her I'm painful urination and so on. And she just said it was down, like I said it was down to my surgery, and it will wear off. That was awful |
| | | | I mean I I understand, you know, people are busy and so on, but you're dealing with someone's life. We should know more and you should listen |
| | | F-50 | It would be nice if there was a leaflet or something to explain things to you rather than, you know, just kind of being treated like just a number and off you go and you know, so I've I've experienced that side of things where you know, I've felt a little bit alone and you know and every time I see a doctor or a consultant, they always say to me, oh, you know, you're an unusual case |
| | | F-58 | your average urologist, I'm afraid, is dismissive and comes up with really barbaric suggestions of what to do to improve you |
| | | | he said to me, well, it's obvious you don't have an infection, I suggest you go and see a psychiatrist, which was so demeaning. And so so awful… I wrote to the urologist and I said, I think you'd need to know that you were really dismissive, you were really arrogant and you were really unpleasant to me and I think the next woman that comes and sits in front of you, sobbing should be treated with a little bit more empathy. You know, I'm a patient. I'm not a textbook case |

| | | | |
|---|---|---|---|
| | | F-48(1) | A doctor came in and said, you know what's been wrong with you and I said I had sepsis and he said you didn't just have sepsis you had septecemia. I said oh OK, I thought they were same. And then I said well what about the other thing, ohh that's fine, you're OK, and I never got any information |
| | | | I'm thinking more you know, what have I got what, what's going on, and then you're getting told oh you're OK. It's not good enough . Very very poor |
| | | F-61 | I know GP says they've got a difficult job to do. Uhm, but because I am a a frequent. Unfortunately, UTI sufferers you you tend to find that dumb. They almost it's kind of I owe you again and and and you start getting lectures on how many antibiotics you're taking and you've got to bring your antibiotic consumption down. As if I'm doing something wrong and and the latest. Kind of. Approach from GPS is try to leave your symptoms for as long as possible so that your your good bugs can try to get on with fighting |
| | | | I always feel that. Mostly, I'm almost asking for something I shouldn't be asking for, particularly in recent years, despite the fact that I am clinically unwell and I've had a positive pressure almost every time. You have to convince them that you're you're ill enough |
| | | F-48(2) | I felt really, really, really unwell, and it took a long time to get somebody to actually listen to me |
| | | | I'm sure I've got an infection and they were saying they haven't and then giving me drugs that I didn't think were the right drugs |
| | | | I said I really, really think I've got an infection and and I don't think like the antibiotics have worked and they they said to me you've just got a bad hip and it's your age |
| | | | I then went back and kind of said OK my understanding is. I really, really think I have got infections. Could you please trust me? And I think we need to be like thinking about whether or not I'm having the right drugs. And then he said, no, it's at your age |
| | | | sometimes when it's really bad. You know, and I and I say to them, it's back. I know it's back and they're like, no, it's not |
| | Having to Fight Alone as a Self-Steward | F-40 | I told him I've done some research and I'm hoping to go and see [specialist] |
| | | | Nothing has been explained to me. I'm just doing my research and understanding now. I could have a resistant UTI infection that was caused by the catheter during the operation and whatever bacteria was brought into the operating table |
| | | | she said 'cause, you know you don't you don't really know a lot. Even some of the answers that I've tried to seek online are not there. So I think they what, what, what what would be the you know how would I get off of antibiotics |
| | | | I have no idea what's going on with me half the time |
| | | | I was reading studies, you know, like late at night whilst I was in hospital or just trying to understand, you know. And I 'cause no one tells you anything in there |
| | | | So it's it's really about the education for me and the stewardship and to make sure patients are heard. So you're in the right dose, the right antibiotic for the right amount of time. And then if it doesn't work that there's no gap like for me I had gaps. And whilst the infection was just taking hold of my bladder and, you know, creating biofilms, as I understand it now |
| | | | what I find extraordinary through the pandemic you know, everyday they're recording how many deaths and then of course, over the year, and like I said to some people, do you realize sepsis has killed more people? And they're like, |

| | | | |
|---|---|---|---|
| | | | what? And I said, yeah, most of those cases believe it not would have been UTIs, you know. So the reality when you kind of explain it |
| | | | you start to become your own expert really in this because you just so desperately wanted to get better. And when I looked at the guidelines for prescribing antibiotics for urosepsis against the abdominal sepsis, the hospital trusts I was at, in fact, if they had listened and even read the ambulance notes that I should have been on a longer course of gentamicin or something else, for longer than just five days I should have received 14 days |
| | | | I just got my help really from all different sources all my information from ANTRUK. Reading other people's stories, speaking to other patients like myself, understanding their journey. Everywhere really. Newspaper, online, from studies I've read lots of studies from around the world, you know, medical papers |
| | | | Reading everything you can possibly about you know what you're going through, and again 'cause you try and find a cure which I know is not out there for me, unfortunately |
| | | | I I think I've absorbed so much of it is I so want to get better you know, I think I've exhausted my research now. I've exhausted the amount of information I could, you know ever be given or found or whatever. I think it's just a a miracle I'm looking for [laughs] |
| | | | I had to make my own way. Yeah, and create my own journey to to get help, you know, which I did from help lines |
| | | | thank goodness got an appointment for February and that's um. Well, it's where another journey started, I suppose. Feeling more secure |
| | | | also to know when I'm getting close to running out because you know, I'm actually quite close are running out of amoxicillin now and I'm like, oh, gosh, I've got two now, you know, write to my GP 'cause, it's very easy to take, take take and then you know, they forget |
| | | | it's OK bringing in new antibiotics, but as I found in my case, it's the stewardship of it and it's listening to the patient. I wasn't listened to in hospital |
| | | | So it's it's really about the education for me and the stewardship and to make sure patients are heard. So you're in the right dose, the right antibiotic for the right amount of time. And then if it doesn't work that there's no gap like for me I had gaps |
| | | | But if you're not listened to, if it's not prescribed correctly, if it's not given to you for the correct amount of time, the correct dose or whatever, then you're not gonna have a chance in hell in avoiding a resistant infection |
| | | | whenever we go on holiday now I have to research where the hospitals are up to make sure I'm at least an hours distance driving distance to a hospital and 'cause I'm just paranoid now that you know something terrible is going to happen |
| | | F-50 | if I have an infection I just carry it on onto the next one or I'll just take more of the one that I'm already on |
| | | | it's made me very, very anxious and I never want to go out on my own. And in case it happens, I'm I'm very scared now of driving by myself because I have been in the car when it's just come on all of a sudden and then I've been like mad full trying to get home in time, you know. And I mean, I've learned now myself kind of how to control it myself |

| | | | |
|---|---|---|---|
| | | | they don't really know. I'm teaching them something, so it is a lot of it is a lack of knowledge and communication and if there was more of that, I think in the earlier days I would have been more aware of what was happening to my body, you know, and my life is in their hands |
| | | | I would say I am teaching the GP side of it, whenever I have an appointment and if it's a different GP if I've been diagnosed with something else, I always say to them, will that affect the rest of my medication? And when they see I'm on antibiotics I'm on they'll query that. And so, you know, and then I end up telling them |
| | | | it wasn't until I started talking to family members and friends, and every time I said to them, I'm on antibiotics all the time now, they were really shocked and they said, Oh well, you know, that really can't be good for you. And so I started researching it a little bit. I didn't really find much, to be honest, and to be perfectly honest with you, I'm not very good with computers |
| | | | I did as much research as I could and then I had another appointment with my consultant |
| | | | I do think, you know, the side effects should be highlighted as well. I know you get when you get your medication, you've got your leaflet and it says, you know, common side effects, but everybody is different and you know, and it doesn't always have that side effects that you're having down there. And then you think oh is this normal |
| | | F-58 | It is absolutely shocking that otherwise healthy people have to suffer like this, you know, and I I can't say that more strongly. I will do whatever it takes to get change affected |
| | | | I will look forward any information I need to forward. I will speak to people like you. Any survey that's taking place, any contact I actually contacted Baroness [anonymous] recently. She was the lady who was involved with the sling, the Mesh campaign and was so successful. Unfortunately, she hasn't got time to take this issue on board, but I will do whatever I can to to affect some change and get better, better outcomes for people like me |
| | | | I've done an awful lot of my own research, to be honest, and it's and it's really Facebook groups and fellow sufferers who've given me most information |
| | | | I'll be honest with you, the every single test I've had done on the NHS has come back negative and I know full well that I have an infection |
| | | | I went two urologist who basically said to me, look you haven't had an infection all these months. This was about September. I said well, you know I'm the patient. I'm telling you, I don't feel right even though you tell me I on paper I haven't gotten infection. So that's how it all started and I've been battling the same thing ever since. |
| | | | you see a cartoon which says, you know, on the patient, I've lived with this for so many years. You're the doctor. You've studied it for an hour on your degree, course and. And that's how I really feel strongly about that |
| | | | I think I know everything is to know about this infection. I've I've I've on I've unturned every single stone. Because that sort of person I am, I won't rest until I get better |
| | | | What else can you think? Oh, I'm fine. I'm not fine. I'm really poorly. And you can't see anything. That's the trouble. It's invisible . And I think you know, the only only consolation to me is that I'm not alone, that there are lots and lots of people, and I'm I'm helping to try and campaign to get awareness of this |

| | | | |
|---|---|---|---|
| | | F-48(1) | There's just no information, and then you discharged you go home and you just left with it, you know. And like I said, I've had to do my own research and calling and trying to find out about ESBL |
| | | | just getting to grips with it all, you know in your mind and You know you just sit back and think, gosh, I've just gone through and but you know what's happened in my body and why it happened. And then you think you know where did I pick it up from? Why did I catch it? I'm always clean in hospital, you know, I was doing my hands, but why did I still get it? I just don't know |
| | | | I think the main thing is you know what will be on people's pain just like it was with me. Firstly, how could I get it? What is it gonna do to my body and what are the symptoms or side effects? And what about medication to treat it? You know, or even to get rid of it. You know, if I could take a medication to get rid of. It I would. You know, uh, 'cause I've got this for life |
| | | | I Would like to know more about antibiotics that are available if if I got it again and I was given Meropenem and if that didn't work, what else is there out there for me? |
| | | | , you just dread getting an infection in case, 'cause I can always tell that I've got, I've got one… I was saying to [charity patient support services], when you've got the ESBL in urine, it's got this certain smell. I can't. Explain it, but if I got it again, I would know from the smell and how frothy and bubbly is when you have a wee it’s got a distinctive smell |
| | | | I’m well in tune with my body I know when there’s something not right |
| | | | I got got good relationship with my GP. We're really good but ESBL’s never mentioned or you know, even like when I've got urine infection and I take it up there and we test it and then so we'll send it away. And that's it. So this is why I have to be like my own doctor, been trained with my body and know myself when something’s not right |
| | | | I just try and live day by day and think if it happens, it happens, you know. I know to get to the hospital and say to them, you know, would you check ESBL if it's I say, woken up |
| | | | I've got a lot going on with health in my life and I just think you know live day by day. I look out for the symptoms now and I know just to get A&E , if I became poorly because it, like I said, it comes on very very quick |
| | | | I don't worry and stress over it, it is what it is and you just ge on with it, I’m that type of person you know |
| | | | I always make sure that you know the toilets bathroom anywhere that I've touched is kept clean, antibacterial and things. And you know I don't overdo it. Yeah, 'cause it sends you crazy. You know I did in the beginning when I didn't know what was going on and then I'm more relaxed now |
| | | | If it gets too bad, I know to go to my GP or if. I've got really. Big temperatures and things I’d go to A&E even though I don’t want to I do look after my health that way |
| | | | It makes me look like when you go to the GP or take this antibiotic, unless it's necessary. Now I say no. Well, I I don't want antibiotics for everything. You know I don’t care if you’re having to change them got puts you on stronger ones |
| | | F-52 | I've kind of learned to tell the difference now that they now trust me that also no, it's not an infection |
| | | | It's that balancing act of trying to get rid of the infection, but also having to tolerate the antibiotics as well. |

| | | | |
|---|---|---|---|
| | | | I have a small sticker at home so I could just swap it and drop it in other drugs when I need so. Yeah, I've got special dressings that, uh, got. It's gold or silver inside there, so it's kind of tries to drag out any infection |
| | | | you do worry about constantly getting infections and stuff. You also have to try and live a life starting this well. So it's that balancing act and then it's being on more antibiotics |
| | | | I volunteer for antibiotic research UK. I've realized that different antibiotics do different things nowadays |
| | | | Even I didn't know, I thought it was my body got used antibiotics and it was only through me volunteering with antibiotic research UK, I've learned that it's the bacterial infection that's become resistant |
| | | | I think apps have made a massive difference. Oh my. The information is there a more infections and different things like that and that's made it a lot easier |
| | | F-61 | Now I tried to look at recent research and I think there's a web MD that I could remember that I try. You know? Then it's not that informative, but. You know, Google you know is richer cricoli and then you get the antibiotics that come up for it and then look at those antibiotics you want. Any more information about them and. And I've I've done. There's a website. Now what was that called? I think it's called chronic UTI. I that was very interesting about new research that's coming about |
| | | | and trying to become. As informed as I can, given that it's something that dominates my entire. Life, you know I. Can't just roll over and play dead and let it win. At least I can be informed |
| | | | So I'm having to fight. I'm having to fight like crazy and to stay well enough to look after him |
| | | | so I have a battle. All the time. To be allowed. To be left with antibiotics despite the. Despite my history and and yeah, I feel like I have to battle to stay well |
| | | | if I do need intermittent oral antibiotics, I have to go. I have to. Go through the whole GP. Up some battle through the receptionist, get speak to a doctor. Gets more antibiotics and this is all just to stay functioning. And you know, I don't really feel I've been given any information or really much help. I'm I'm on my own battling alone |
| | | | A sense of being cared for would be more help and a sense of continuity of care would be more help. Uhm, because Honestly, I feel that with every episode whenever infection episode, I have to start at the beginning and I have to work like the devil to get through to the doctor's surgery to try to have a conversation with the doctor and to try to get antibiotics in time and and it's. Such an effort when you're not feeling well |
| | | | I always have to ask for the standby antibiotics. So as I say there's there's never a case of. How can we make this easier for you? It's always me having to do. All the push all the hard work |
| | | F-48(2) | I understand sort of battling this. I'm sure I've got an infection |
| | | | my family find it really difficult as to my friends and I think partly is 'cause. They want to help, but they also just. I don't understand how I can continuously either be fighting for tests, getting test results, waiting for test results, having an infection, taking other drugs |
| | | | I kind of do worry about that a bit. I do worry about, kind of. Well, I don't really and I don't really understand how it all works, you know. I've done. A lot of reading trying to sort of broaden my own understanding to be able to ask kind of intelligent. And I I'm really limited in. No, this is repetition. But in terms of. What I can do |

| | | | |
|---|---|---|---|
| | | | I was researching the Cipro factor for indeed antibiotic resistance impact for Indy. Uhm, you know pathogens in there. Thing, and there was some really good, UM. Not interesting, I've got some really interesting research that had been done that was published in the Asian sort. It's a general bit like the BMJ top thing around using eucalyptus and eucalyptus leaf specifically for Enterococcus E. Col |
| | | | the specialist from this morning they were like you know more about this than we do, 'cause it's your body |
| | | | I think if I couldn't advocate and I was an older person or something, I'd Probably be dead |
| | | | I think stuff around how to navigate and deal with healthcare professionals, so about how to communicate |
| | | | They would then like issue something and I'd say Are you sure that it's going to be sensitive to this, you know, and they were like, Oh yeah, well, you know it be fine, you just need to take. It just needs to take it and then three days later they drink 'cause they've got the results back their own internal. Don't say that anymore. You know, take something else. I'm like you are messing around with my body here. So I didn't. Basically I didn't take it so they'd like give me, you know, this sort of standard thing you'd give for a UTI, and I thought you know what until we've got the lab results. Back I am not even going to try it because all it's doing is basically the more entry point mark |
| | | | from my layperson I'm not a medical person. Understanding it looks like that infection isn't sensitive to those. Drugs from say like the right way around. You know, and that was the start of me trying to understand more about what was going on |
| | | M-78 | I've studied in-depth. As I say, I'm fully computer literate and I work things out for other people as well, you know. It's just something that I do, so I look all these things. They helped out there. What's the, what's the word? Thinking about all the diseases that I've read about, you know, I just read it for information purposes, and this is what I've got in front of you now about the about the different sites. |
| | | | when the doctor suspected osteomyelitis- I've never hardly heard of osteomyelitis, so as soon as she suspected that and sent it for the X-ray, that's when I look up on it all, you know, and you push, there's masses and masses of stuff on all these different subjects on the internet, as you know. You know you got to be careful because some of them conflict with others. It's, you know, I was reading about, I've been reading about herbs herbal remedies have the different things because COPD I was looking at the herbal remedy, the COPD, I was looking at one of them. And how soon does that, does that interact with all things cause most forms interact with all? Some worse than others and also things like cranberry juice and obviously greens and things like that. |
| | | | I looked to see if alcohol effects with particular, and one said there's no interaction. […] And then another one said that this. You're gonna be very careful when. You read up on these. |
| | | | So you gotta make sure you read the right ones, but there's there's a lot of ones in America, American websites, that's medical sites that are very good. And I've got a list now of all the interactions, all of the drugs that interact […] a lot of people, they don't read the leaflet, they don't read the leaflets to see that they should be keeping their drugs at less than 25 degrees. We're getting days when we got up to 35 degrees, coming up and have been in the past, you know, so |

| | | | |
|---|---|---|---|
| | | | you gotta watch for things like that and also what they interact with and also, your diet. |
| | | | If you read up on them, you could become a lot more wiser. |
| | | | by reading half a dozen and seeing the general consensus, you know you will get, you'll get poor and say that something's OK and to say that the opposite or not quite the same, you know, so you would obviously, I know. The best sites to go to as well. Now you get some that are, you know, trying to fill your stuff all the time and you get other ones that are genuine, genuine medical sites, you know. And you get to know the difference; you know, they know what they're talking about. |
| | | | they knew that I was familiar with the condition, and what was occurring as opposed to most of the people […] other people don't have computers or look things up. It's unable to, you know. I'm very lucky in that respect that I'm self-sufficient. |
| | | | No, I have not asked for it [support]. […] I'm one of those people that can cope. You know, I'm 78 years of age and I've been about. |
| | | | I do my feet everyday with the antibiotic biological wipes that we use everyday that we see everywhere. You know, I use that because it dries very quickly. […] So it's like a liquid spirit, like alcohol. And that drives very quickly. And then once that's dried and I put the spaces in between my toes and just try and check and then the nurse checks as well to make sure that the sores aren't coming back again because they can come back very quickly. |
| | | | I think people have got to apply themselves more. They tend to take what they're told and accept it, which is the wrong, I think. If you need something done you've got to make sure you get it done, not just get told to go away, it will be alright as is the case a lot of the time. |
| | | | You gotta be persistent with your doctor. You must make sure that you get asked them […] you gotta ask for it and it's just like. So that's the sort of thing I will pass on to other people. |
| | | | You're not being that rude to your doctor. You've got to be persistent because you just won't get things done otherwise. And this of course applies with anything, you're trying to get a refund on something- you bought the shop and they try and pull me off. You don't accept it, you know. You're gonna take the other thing, or you're gonna, you're gonna write the papers or whatever, you know. You've got to be persistent. |
| | | | You've gotta be the same with your doctors, you've got to, which is what I've been doing all this time in order to get this. […] Otherwise, I would be sitting here like this and not knowing I've got it and that could then spread, you know. |
| | | | It's just applying yourself all the time. You know what I'm saying? People are not used to doing that unfortunately, so I don't know what I could say to help them. |
| | | | What I'm telling you, to apply themselves more and be more persistent to get what they want, you know. Without being rude and over the top. I'm talking about if you've got a particular concern, I mean, if you have reason to think you've got cancer and you want it sorted out, wouldn't you? You know the same. As I've got a concern that I might have lost during my life that I'm getting it sorted out. It's taken some time that, that's not my fault. That's only because people just haven't taken any notice of my request until now. You know, and that was and that was done. Not by A&E. Which they should have done. |
| | | M-26 | I think if I was given information about the actual extent of my condition at the initial point in time, that would totally like help me to talk about this. |

| | | | |
|---|---|---|---|
| | | | Well, um I would actually like to get updated information about this condition that I'm having that so everyone can know about the medications and techniques. |
| | Feeling Supported by Clinicians | M-78 | The doctors have been very supportive and the consultant about the letter. […] get the consultant to write a letter to them, and also then you're a doctor to write a letter to, to take to A&E and to be seen immediately in A&E. No waiting at all to go up and get put on the bed. And for the for the vascular team to come and see you all within an hour. From the writing. This was 10 times, 100 times better than my local hospital. |
| | | | So when I took the letter there, they took notice and I was scene within an hour by the team. That's incredible service, you know, like I was royalty, you know. I choose the vascular team. Come and meet me in a wheelchair and push me up to a wall. A beautiful reclining bed with all the services it's trying to private them. Quite incredible. |
| | | | I've been very lucky with the nurses, very lucky indeed. We've got our doctors- we've got 5 surgeons. And the main one is for me to park and walk to. There's very limited space there, close to the surgery. Where I can go to, I can go to the. Four or five chargers that I got parked outside, and so I've been able to go three times a week to see the nurse. At the moment, I'm going to one particular surgery and see the same nurse, and she's very, very good and she also takes notice of my walking, and I'm seeing her every Wednesday. So she's been looking at my foot and […] has been checking to make sure that the infection is not returning because my feet, because my toes are pushing together. |
| | | | They're there if you if you need them, but I haven't requested any particular support in that area. Don't forget I've seen been seen in those three times a week up until recently. So that that's been a great- they've all been very supportive, and I get on very well with the one that I see on a Wednesday. They shall, she's very- […] even like the next appointment, you know, because she's having a chat. So she's very supportive |
| | | | help each other which is great. So that's, I only have to see her once a week now […] to take my iron and just to check my toes, you know. So that's great ongoing support you know. |
| | | M-26 | All the information that they gave to me about it, was that I had to be very active on my medications because the infection that I had was very resistant to wide range of antibiotics, and I had to keep a lot of checks on my health and I had to do, I had to. You know, take my medication serious and make sure that I finish them off when I was sent home to use them. That was helpful. |
| | | | my experience is OK, that's been quite OK because I got, I've been able to get less. I think that's the only place where I kind of, you know, feel free to talk a lot about this challenge that I'm having and then I think I feel a lot more relaxed when I'm around. So it's more I think they can have more of an idea and you know understand my condition more. |
| | | | Yeah, so I feel more supported by healthcare professionals. |
| | | | Yeah, it's, it's, it's hospital and they've been very supportive. |
| | | | I feel that it's helped me. I feel that it's helped me a lot. I think that's where I can get support from so that's been helpful. |
| | | | Yeah, I think the, you know, they provided a lot of online resources where I could learn a lot more about this condition. |
| | | | Yeah, I was given information and advice about medications. |
| | | F-50 | I absolutely love walking and she gets me up and I go for a lovely long walk and I clear my head while I'm walking. Uh, I get, I try and get energized from it. Uh, |

| | | | |
|---|---|---|---|
| | Exercise, Activity and Focus | | I'll try and I've started doing Pilates, I've tried meditation, I've tried all like the mindfulness side of things to try and get focused |
| | | | if I'm feeling too poorly to get out of bed, I will, you know, really force myself even if it just means sitting on the sofa downstairs under a blanket, you know, I really hate spending the whole day in bed because I've spent far too many days in bed being ill |
| **(Theme 3)** I like to share my story: The role of AMR communities | Accessing Information Via AMR Communities | F-40 | I've got to thank the sepsis UK trust for that, because if I I, I rang them about 20 times for advice because I was so worried and 'cause no one can help |
| | | | she's really enlightened me in how serious UTI are and it's a little pandemic going on really you know, that about 50% of the phone call she gets or the enquiries are from people like me |
| | | | I met someone via ANTRUK, and it's a guy in India, and he has had phage therapy so he's the one that's got me thinking about it |
| | | | I just got my help really from all different sources all my information from ANTRUK. Reading other people's stories, speaking to other patients like myself, understanding their journey. Everywhere really. Newspaper, online, from studies I've read lots of studies from around the world, you know, medical papers |
| | | | it's just through ANTRUK, really, Arlene has become like a friend, a mentor. I mean, how does she do it? |
| | | | I learnt a lot from just talking to people on in over the help lines and so on, getting involved with things, speaking to other people in the same boat. I think there's Facebook groups as well that ANTRUK has developed, so you can speak to people in the same kind of situation. |
| | | | At the Sepsis UK trust, absolutely fantastic. They alerted me to the fact I could have a resistant infection in the first place |
| | | | I think the best help I've had is, is just from organizations that are there specially for sepsis, specially for resistant infection. And talking to other people so you don't feel alone, so you can share your grief, share your experience, share how you feel with other people that are going through it, you know |
| | | | It was nice to talk to someone, but again, they're not in the know how how it feels or or you know involved in it in that way |
| | | F-50 | I researched it all myself and I come across the charity antibiotics research UK and they had just started to pull a support group together and then Arlene there who I chatted to and I'll text to and I email to, she is being absolutely brilliant and her knowledge is fantastic and she's basically has been my support person and has given me all the information I need to know she's been reassuring |
| | | | I have through the charity met other people that are on antibiotics and suffered previous instances and I've managed to talk to other people about it, which is great. And that's, you know, my way forward. If I can talk about something and understand through talking |
| | | | I don't know what it is about that woman, but she just she just makes you feel you know that your life is worth living, basically, and that she understands, she understands what's going on. So yeah, she she's been the most helpful person in all of this, I would say |
| | | | She's put me in touch with other people, we have zoom meetings. It's really, yeah, it really has been the support side of it that I've needed, I think uh. I think to begin with I felt so alone, but knowing now that there's thousands of people you know, not necessarily with my infection, but with, you know, fighting with these infections and having resistance and which you know is really scary to live with. But talking to other people that have it, you get little tips off of them you |

| | | | |
|---|---|---|---|
| | | | know, how do they live their lives daily, you know? And you might think, oh, why I didn't think of that, you know, so little things can really help, but I think, yeah, the best, best side of it always is talking to people about it |
| | | F-58 | I've done an awful lot of my own research, to be honest, and it's and it's really Facebook groups and fellow sufferers who've given me most information |
| | | | primarily it's it's patient support groups without a shadow of a doubt because you could have an open and frank conversation and people will say have you tried this particular supplement or ohh when I when you're taking this antibiotic makes sure that you do it after a meal or don't lie down immediately afterwards. You know things that you'll probably be able to read on the packet. But I've had a lot of useful advice from patient support groups and that's been my main source of information |
| | | F-48(1) | I just went home and I had an appointment with Urology and I spoke to one of the nurses there and she said to go on the Internet. And that's where I got in touch with [charity patient support services], and I got all my information |
| | | | I just I just speak to [charity patient support services]. Yeah, I bring it her and I get a lot of main information from her. I go on the website and we look at people stories and you know and that's it really |
| | | | I looked up the people stories and what they've gone through and some with urine infections, you know, and how they've dealt with it and how they found out and just looking up more about what the ESBL is now, it's spread and what it can do to your body and how it can make you ill, and you know that they can kill you these superbugs |
| | | F-52 | Even I didn't know, I thought it was my body got used antibiotics and it was only through me volunteering with antibiotic research UK, I've learned that it's the bacterial infection that's become resistant |
| | | F-61 | it has been so uplifting knowing that there's somebody somewhere trying to do something |
| | | | considering she's working for a charity, I can't tell you how much support she's given me. It's it's been amazing |
| | | F-48(2) | So in terms of the only real information that I've had from anybody that really knew what they were talking about was Arlene from the antibiotic resistance support thing |
| | | | The most useful conversation that I had was with Arlene from antibiotic Resistance UK and she set up. There was like a meeting like a little meeting. And and there was our lead myself, and there was a woman who was in Dubai who was from America. It was on zoom. Uhm, and that was probably the most informative hour I've had from this whole subject |
| | | M-26 | We have access, we have, I think the first providing this service and providing this for and then I I was, I was going to support organisations for my condition. |
| | Emotional Support Via AMR Communities | F-50 | we chatted all about it. How you know this infection is ruling my life now, but the tablets are actually saving me. Uh, and we explained to them that, you know, there could become a time when the tablets will stop working, and if I get my infection, you know, this could become quite serious |
| | | F-58 | it's sort of CBT sort of talking therapy, but it just gives me a space. It gives me somebody who listen once a week |
| | | | I speak to other women in the same situation, which gives a bit of comfort |
| | | | I speak to other sufferers as well. You know, you can get a bit obsessed with it and you have to step away sometimes just to try and have a a day when you're not thinking about this condition as best you can |

| | | | |
|---|---|---|---|
| | | F-48(1) | but I think the people that. Are living with it. Are the best people to tell their stories and how it's affected them and what happened to them. For people to watch and listen and be interested in it |
| | | F-52 | that is hard when you see friends came and sat down and all that and it is harder and having. To talk about that. Is good, but you know I feel like in different ways, yeah and. Doesn't even just massively help your mental health, and I feel like I'm giving back as well |
| | | F-48(2) | They might not have the answers, but they understand what it can be like to live that way. 'cause I guess they've just seen other patients in that situation. So if somebody understanding having some empathy, even if they don't have a solution. Is an incredible gift |
| | | | they've been able to talk to somebody who was having who had had a similar, was having a similar experience. Uhm, but not having to explain every single thing and not being asked like really stupid pointless questions. Because I was. So sorry, that sounds really awful, doesn't it? I've been asked 8 million times, so being able to, yeah it's being able to talk in a safe space with with with somebody who was. Uhm, and focused on. Practical things you know, like it's important |
| | | | I think a group would be quite good if it was, you know there was space to talk, but it was structured and facilitated. Uhm, rather than like. And there was like information. If you know what? I mean like. Maybe practical, safe space with a little bit of maybe some emotional support or side support people |
| | Becoming an Advocate for Others | F-40 | I feel like I mean, uh, so kind of a support bubble. I said I would say and Facebook I, you know, I go on sometimes and I find that I help others on there, no one helps me. And I suppose 'cause of the role I have with ANTRUK, I end up [coughs] sorry I end up helping other people. You know, no one really understands, you know, kind of like what's happening with myself. So I find that I'm the support |
| | | F-50 | now, my goal in life is to help people with what I can tell them. And so yeah, she's kind of given me a purpose, I suppose in life again 'cause I was at home not working, not really doing much apart from being ill and worrying about it all. So she's, yeah, she's giving me kind of a purpose in life again, and educated me about antibiotics and educated me about the resistance side of it all as well |
| | | F-48(1) | I find it quite interesting, you know, and I I love looking up and reading about it and knowing you know, getting that knowledge. And information. And then when I go into hospital like I said, I end up having to explain to the Nurses, what it is |
| | | | I even speak to friends and family about antibiotics. You know, and I think, do you really need them? You know there’s a lot of things that you can do before antibiotics, obviously if it's something serious but not for silly things like, uh, you know, an inflamed throat or things like that, you know I think we all have to stop and think about what we're all doing because so many people now are becoming resistant to antibiotics cause they take them that much and you know it's going to be difficult for our children or their children in the future |
| | | | This is why I like talking about it and helping and you know for others to get the knowledge about how serious they are, because a lot of people don't take these seriously |
| | | | This is why I like to tell my story and and to help other people, knowing that you know, yeah, you've got this. There is medication, antibiotics, but you know you you're not going to give it to your friends, family using good hygiene and you know if you go into hospital, they're going to treat you ok, not gonna be. Treating you like a lepper, you know you might have to go in a room of. Your own, which isn’t the end of the world |

| | | | |
|---|---|---|---|
| | | | I'm always speaking to people about it the ESBL and they're quite shocked, they think they always say the same, what is it? And I say have you never heard of it and they say, can we catch it off you and you know and I sit and explain to them, and they're OK afterwards, but they're quite interested to hear of a, you know, hospital super bug that's never mentioned to them you've never heard of |
| | | F-52 | I think through volunteering I've become a better patient kind and and I think that comes of age as well |
| | | | I volunteer for antibiotic research UK. I've realized that different antibiotics do different things nowadays |
| | | | I can actually tell people because you know people don't understand it, I've never heard of it before, but that's the case of any illness that people don't really know much about until it happens |
| | | | I'm hoping that I'm sharing the knowledge of antibiotics and antibiotic resistant infections do happen to people |
| | | | My friend just had a section C-section rather than a baby and she thinks her scars infected and I'm trying to get it through. To her … Go get checked out. But it's like it happened to me, but still this infection |
| | | F-61 | She's given me information on on. On the bug itself, and when I said it was only Google, Arlene has talked to me about it, talked to me about other complementary things that I might. Be able to do. And probiotics and things like that. And and just being very gentle and kind and listening to me and understanding and and helping me to perhaps be a little bit stronger in asking for things with the GP and the hospital and and. And that has has really helped |
| | Charity Based Support | F-61 | the only people who have supported us have been the Maggie Center, which is a charity, and St Columbus Hospice, which is the charity and the brain tumor charity which Jamie is raising money for at the moment. Yeah, are the only people who the biggest funder of research is astonishing that it is charities that are helping people, not not the government |
| | Support Via Family | F-50 | We've had to really, uh, learn how to live differently. You know, I mean, I I don't work, I had to give up my work |
| | | | he's completely turned his life upside down to be my carer basically |
| | | | my mom lived in Turkey at the time when I was very ill, and she's actually moved back to the UK so that she can be here for me. So she's had a massive change in her life as well. So, you know, I mean, massive things like that have happened |
| | | | we had a code system in our house once where we would have like a a white board up with like a green, amber and red, and for the children's sake and for my husband if I woke up and I wasn't feeling too good, I would tick the amber box. If I was feeling really rough I'd tick the red box so that they knew to be alert. And and you know, we did do things like that |
| | | | I'm lucky I get the support from my family, you know, not everybody has that |
| | | F-48(1) | my husband quite understanding and once we've got the information and I've explained everything to him |
| | | M-26 | Well, I think I really with this condition my mom has actually been very active, and you know, keeping tabs on me and checking up on me with this condition. That's just, that's just fine. Fine. |
| | Family Distress | F-50 | I have to say not just for me, for my family as well. I mean, my poor daughter she's growing up with, you know, ambulances, seeing me, lips go blue, shivering, not being able to move. And, you know, it's been awful for her |
| | | | I think for them and anytime I say, oh God, I'm really cold there, like, Oh my God, are you OK? You know, they're on edge all the time |
| | | | My daughter, she's had to have therapy because of it, it's affected her |

| | | | |
|---|---|---|---|
| | | F-58 | the most difficult thing about all of this is my relationship with my husband because. You know, it would be married nearly 30 years and he's he's a good man and he's so he's, you know, he really does his best, but he doesn't know what to say anymore. And I feel, you know, my life has shrunk and his life has shrunk as well. And I'm I'm. I'm tearful, I'm unhappy I'm I don't want to be needy but I am and you know the whole balance of our relationship has shifted because I was always the one who was in control and organizing things organising social life |
| | | | My daughter's gonna get engaged soon and that you know, it should be a really happy time. But. [participant is tearful] I don't know if I'm going to be able to manage it. You know? How do I go and look for a dress with her? |
| | | | my husband, you know, we can't have any sexual relations that's just gone completely out of the window. And as I said, I I don't want to be this needy person, you know? And and sometimes he just has to go out. He just has to leave the house for a couple of hours a night. I totally understand that. But that makes me feel even more guilty |
| | | F-61 | it's a desperate, desperate situation. So yeah, I don't like to lean on on the boys too much, but they do know and they are aware and they are actually very worried now about me as well as their father |
| | | | the impact it has had on. My husband has been palpable. He has worried for years about my my health |
| | Support Via Friends | F-50 | my true friends that I see all the time, she's actually researched it herself to try and understand me a lot better |
| | | M-78 | my friends know me very well and there's no there's no problem there |
| | | | they'll know. It's obviously a topic of conversation that I limit to just a small amount of time […] But they're interested to know how I'm getting on. |
| | | | I've got loads of friends […] Yeah, you can trust him with your life, you know, very generous person, yeah. |
| | | | To answer your question, no, I don't really need myself to go to a group because of the interaction I get with my friends and each other, you know. My friend that has got a problem down below […] and I take him up to the hospital and things like that. So again, we support each other he's a little bit older than me. We support each other you know in that respect, with you know, with genuine concern |
