# Supplementary material for: Understanding the lived-experience and support-needs of people living with antimicrobial resistance in the UK through interpretative phenomenological analysis
Source: Sci Rep. 2024 Feb 10;14:3403. doi: 10.1038/s41598-024-53814-6 (PMC10858033; doi:10.1038/s41598-024-53814-6)
Supplement: Supplementary file 2 — Supplementary Information. [file 41598_2024_53814_MOESM2_ESM.pdf]

Interview Schedule

Note: Slight amendments to the wording of these questions/prompts were made during the interview, based on the responses of the participant

Introduction

- Welcome, introductions, and initial rapport
- Taking consent for participation and recording

About the patient

"Can you tell me tell me about the antibiotic resistant infections that you have currently, or had previously?"

Prompt/follow: What microbes were causing your infections?
Prompt/follow: What treatments did you need for this?

"Other than active infections, are you also known to be carrying an antibiotic resistant microbe?"

"How long have you been affected by antimicrobial resistance?"

"How did you find out that you had an antibiotic resistant infection, or were carrying a resistant microbe?"

Prompt/follow: Did anyone tell you about this verbally?
Prompt/follow: Did a health provider, such as the hospital or GP, write to you to inform you of this?
Prompt/follow: What information did you receive when you were informed about this? Did it explain things to you? How useful was this to you?
Prompt/follow: What were you told? What did you understand? Etc.

Experiences of antibiotic resistance

"What have been your experiences of antibiotic resistant infections?"

Prompt/follow: Did anything worry you in particular?

"Can you tell me about other ways antimicrobial resistance has affected you?"

Prompt/follow: Day-to-day management, coping with impact of this.
Prompt/follow: How do you feel antimicrobial resistance has impacted on your overall wellbeing?
Prompt/follow: Do you think antimicrobial resistance has affected your health, other than through infections?
Prompt/follow: Has antibiotic resistance affected relationships with your family and friends? Have you been able to tell family and friends? Reaction? If not, why not? Etc?
Prompt/follow: Do you tell people about your microbial resistance? How do people typically react?

"What are your experiences with regards to healthcare since your diagnosis?"

health/healthcare front, but also holistic overall wellbeing experience
Prompt/follow: Do you feel this has affected the quality of care you are given? For example, do you believe healthcare workers will shy away from giving you the quality of care you deserve?

Support for people affected by antimicrobial resistance

"How do you feel about the information and support you've been given by professionals about antibiotic resistant infections?" (more of an ongoing perspective, not so much that initial info)

> **Prompt/follow**: Were you provided any resources that helped you to better understand your condition?
> **Prompt/follow**: Were you given sufficient advice about your diagnosis by a health professional?
> **Prompt/follow**: Were you given the opportunity to ask questions about the condition?
> **Prompt/follow**: Were you comfortable discussing the diagnoses with the health professional? Were you satisfied with the advice you were given about the diagnosis?
> **Prompt/follow**: Have you received any support from friends and family?

"Are there other support systems which provided reliable information and support for you? What are these and how have they helped you?"

> **Prompt/follow**: If received support through healthcare – how has this helped you? Anything helpful/unhelpful?
> **Prompt/follow**: If accessed patient groups – how has this helped you? Anything helpful/unhelpful?
> **Prompt/follow**: If not used a patient/peer support network – do you think this would be useful? Why useful? Why not accessed this?

"What information do you think people in your situation need?"

> **Prompt/follow**: Is there anything you wish you knew when you were initially diagnosed?
> **Prompt/follow**: You've experienced this for some time, what information/knowledge do you feel you still require to help you?
> **Prompt/follow**: Where would you like to receive this information and support from?
> **Are there any barriers to accessing support**? Could these be overcome?
> **If struggled to tell family/friends** – what would be helpful to help you do this?

Close

"That's all of my questions, is there anything else you would like to discuss?"

"If you do need any further support or information, I'd like to take this opportunity to signpost you to Antibiotic Research UK which has a patient support service and has kindly funded this research"

"I have stopped recording the interview. Thank you for your time and for your open and honest responses."

"Would you like to be contacted after the project has finished with a summary of the findings and any outcomes from the research?"

"Goodbye" (not scripted – will be a natural goodbye)

Participant Demographic Form

Thank you for your interest in taking part in an interview for this research study. This form asks some questions about you and your condition so we can better understand your circumstances before the interview.

Background details

What is your gender?

Please mark with an "X" Male ☐ Female ☐ Other ☐

If other, please tell us which term you prefer ___________________

What is your age in years? _________________

What is your ethnic group?

*Please select **one** group by marking with an "X". These are based on the NHS ethnic categories groups.*

White British	☐	Pakistani	☐
White Irish	☐	Bangladeshi	☐
Other White	☐	Other Asian	☐
White and Black African	☐	Black Caribbean	☐
White and Black Caribbean	☐	Black African	☐
White and Asian	☐	Other Black	☐
Other Mixed	☐	Chinese	☐
Indian	☐	Any other ethnic group	☐

About your health

What type antimicrobial resistant infection do you live with?

Please mark with an "X"

Urinary Tract Infection	☐	Skin and Soft Tissue Infection	☐
Respiratory Infection	☐	Sexually Transmitted Infection	☐
Bloodstream Infection	☐	Other	☐

What is the name of your antimicrobial resistant infection (if known)?

____

In what year did you find out about your antimicrobial resistant infection? ____________

Which medications or treatments did/do you need for your antimicrobial resistant infection?

____
